# Regulatory Roles of Non-Coding RNAs in Colorectal Cancer

**DOI:** 10.3390/ijms160819886

**Published:** 2015-08-21

**Authors:** Jun Wang, Yong-Xi Song, Bin Ma, Jia-Jun Wang, Jing-Xu Sun, Xiao-Wan Chen, Jun-Hua Zhao, Yu-Chong Yang, Zhen-Ning Wang

**Affiliations:** Department of Surgical Oncology and General Surgery, First Hospital of China Medical University, 155 North Nanjing Street, Heping District, Shenyang 110001, China; E-Mails: cmuoscar@163.com (J.W.); songyongxi840309@126.com (Y.-X.S.); mabin0326cmu@163.com (B.M.); wjj100@163.com (J.-J.W.); sun2003999@163.com (J.-X.S.); chenxiaowan1826@163.com (X.-W.C.); zjh900521@163.com (J.-H.Z.); yangyuchong1987@126.com (Y.-C.Y.)

**Keywords:** non-coding RNAs, colorectal cancer, dysregulation, biomarkers, targets

## Abstract

Non-coding RNAs (ncRNAs) have recently gained attention because of their involvement in different biological processes. An increasing number of studies have demonstrated that mutations or abnormal expression of ncRNAs are closely associated with various diseases including cancer. The present review is a comprehensive examination of the aberrant regulation of ncRNAs in colorectal cancer (CRC) and a summary of the current findings on ncRNAs, including long ncRNAs, microRNAs, small interfering RNAs, small nucleolar RNAs, small nuclear RNAs, Piwi-interacting RNAs, and circular RNAs. These ncRNAs might become novel biomarkers and targets as well as potential therapeutic tools for the treatment of CRC in the near future and this review may provide important clues for further research on CRC and for the selection of effective therapeutic targets.

## 1. Introduction

High-throughput approaches revealed that most of the eukaryotic genome can be transcribed, with approximately 20,000–25,000 genes encoding proteins [1], whereas the vast majority of untranslated fractions of the transcriptome are transcribed as ncRNAs. However, functional ncRNAs may arise from only a small fraction of the total genome, and the most optimistic estimates for the number of ncRNAs in the genome from various sources would place these as being transcribed from less than 2% of the genome [2].

These ncRNAs were recently recognized as a novel class of RNA molecules that are involved in the regulation of biological processes, including gene expression; epigenetic processes; cell differentiation, proliferation, migration, and apoptosis; transcriptional regulation; post-transcriptional regulation; organ regeneration; and human diseases [3,4,5,6]. Although these genomes have important biological roles, several controversies exist [2], and further research efforts are necessary to explore the functions of these ncRNAs.

**Figure 1:**
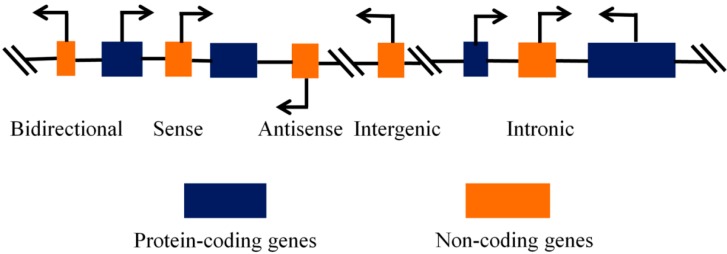
Five categories of lncRNAs. According to the position in the genome, they can generally be classified into the following categories: intergenic, intronic, bidirectional, sense, and antisense lncRNAs.

The ncRNAs can be systematically classified into two groups according to size, namely small ncRNAs (sncRNAs), which are shorter than 200 nucleotides (nt), and long ncRNAs (lncRNAs), which are longer than 200 nt. The sncRNAs can be divided into microRNAs (miRNAs), small interfering RNAs (siRNAs), small nucleolar RNAs (snoRNAs), small nuclear RNAs (snRNAs), PIWI-interacting RNAs (piRNAs), and other sncRNAs. The classification of lncRNAs is based on four major characteristics, namely genomic location and context, effect exerted on DNA sequences, mechanism of action, and their targeting mechanism [7]. In addition, they can generally be classified into the following categories: intergenic, intronic, bidirectional, sense, and antisense lncRNAs [8] (Figure 1).

Colorectal cancer (CRC) is one of main causes of cancer-related death among males and females, and the mortality from CRC is markedly higher in developed countries than in less developed countries. The risk factors for CRC include tobacco use, being overweight, and physical inactivity; however, the mortality from CRC has decreased in many countries because of early CRC screening and improved treatments [9], and the survival rate of patients diagnosed with colon and rectal cancer was 60% or higher between 2005 and 2009 in 22 countries [10]. However, the biological processes and molecular mechanisms of CRC remain unclear.

The dysregulation of ncRNAs has recently gained attention because of its close association with human diseases including cancer. ncRNAs can act as oncogenes or tumor suppressors in CRC, and are potential diagnostic or prognostic biomarkers, with possible clinical applications. In a previous study, we comprehensively summarized current advances in the roles of ncRNAs in gastric cancer [11]. In the present review, we performed a full-scale overview of several ncRNAs and their roles in the development and progression of CRC. This information may provide important clues for further research on CRC and for the selection of effective therapeutic targets.

## 2. MicroRNAs in CRC

MiRNA-lin-4 was first identified in *Caenorhabditis elegans* in 1993 [12]. Since then, an increasing number of miRNAs have attracted attention because of their biological roles in different human diseases, including various cancers. Relevant studies on miRNAs will help clarify the molecular mechanisms underlying their function.

Advances in analytical methods have enabled the identification of several miRNAs involved in CRC. These miRNAs can exhibit dysregulated expression and play important roles in signaling events as oncogenes or tumor suppressors. The gene regulatory role of these miRNAs is mediated by binding to the 3′-untranslated region (3′-UTR) of their target mRNAs, resulting in translational repression. The miRNA-mRNA interactions and their roles in CRC have been studied extensively [13]. Furthermore, these miRNAs may be involved in each process of CRC metastasis, including angiogenesis, invasion, intravasation, circulation, extravasation, and metastatic colonization [14], and serve as potential prognostic or diagnostic markers and therapeutic targets in CRC, and could be developed as therapeutic tools in the future [15,16,17].

### 2.1. Dysregulated Expression of MicroRNAs and Their Putative Roles

#### 2.1.1. Oncogenic miRNAs

**Figure 2:**
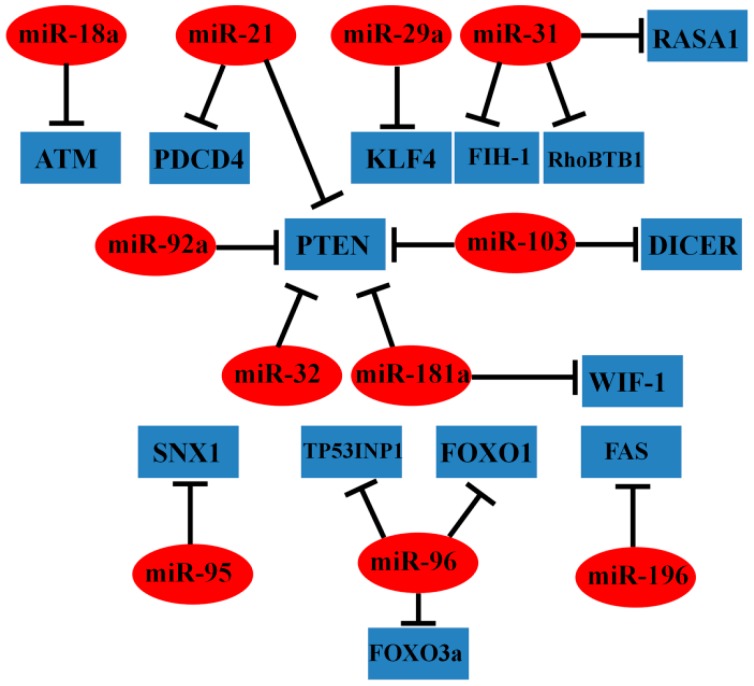
Representative oncogenic miRNAs and their target genes in CRC.

Accumulating evidence suggests that miRNAs are aberrantly expressed in CRC and may serve as oncogenes or tumor suppressors depending on their downstream targets or associated signaling pathways [18]. Certain oncogenic miRNAs, such as miR-18a, -21, -31, and -92a, are involved in the development and progression of CRC (Figure 2).

Aberrant expression of miR-18a, which belongs to the miR-17-92 cluster, has been reported in several cancers such as bladder [19] and pancreatic cancer [20]. miR-18a upregulation was detected in 45 primary rectal tumor tissues compared with adjacent normal tissues. Ataxia telangiectasia mutated (ATM), which was identified as a miR-18a target gene and a key enzyme in the repair of DNA double-strand breaks, is downregulated in CRC tissues and its expression is inversely associated with the levels of miR-18a. miR-18a overexpression significantly inhibited the repair of damaged DNA and enhanced etoposide-induced cell apoptosis. miR-18a was shown to play an oncogenic role in CRC partly through the downregulation of ATM, and it may serve as a potential biomarker for CRC [21].

B7-H1 was shown to contribute to cancer immune evasion by promoting T-cell apoptosis [22]. Zhu *et al.* [23] showed that miR-21 is involved in the suppression of phosphatase and tensin homolog (PTEN), and PTEN expression is negatively correlated with B7-H1 expression, suggesting that miR-21 upregulation in CRC downregulates the expression of PTEN. miR-21 was also shown to be significantly overexpressed in CRC tissues from 30 patients, and knockdown of miR-21 inhibited cell proliferation. PTEN was similarly identified as a target gene of miR-21, and miR-21 regulates the expression of human telomerase reverse transcriptase (hTERT) through the PTEN/ERK1/2 signaling pathway [24]. Meanwhile, PDCD4 was identified as another miR-21 direct target whose expression is inversely correlated with that of miR-21. miR-21 upregulation was only observed in precancerous adenomas, but not in non-tumorigenic polyps from endoscopic samples [25]. Quantitative RT-PCR analysis of samples from a Japanese cohort (stage I–IV) and a German cohort (stage II) showed that miR-21 overexpression in CRC tissues is associated with poor survival in both cohorts, which may estimate the prognostic outcome and identify CRC patients who may benefit from adjuvant chemotherapy [26].

miR-31 was significantly upregulated in 25 pairs of CRC tissues and was negatively correlated with the expression of its target, factor inhibiting HIF-1α (FIH-1), both in tissue samples and in cells. The miR-31/FIH-1 axis was shown to promote CRC cell proliferation, migration, and invasion *in vitro*, and to modulate xenograft tumor growth *in vivo*, and was associated with CRC progression and poor prognosis [27]. This was supported by another study which showed that miR-31 is upregulated in colon cancer tissues and cells, and the suppression of miR-31 inhibits anchorage-independent colony formation and cell proliferation. The tumor suppressor RhoBTB1, a new target gene of miR-31, is downregulated in colon cancer tissues. By targeting RhoBTB1, miR-31 could contribute to the development and progression of colon cancer [28]. An inverse correlation between miR-31 and RAS p21 GTPase activating protein 1 (RASA1) expression was demonstrated *in vivo*, and RASA1 was identified as a novel direct target of miR-31. Furthermore, miR-31 could play an important role in modulating the RAS signaling pathway by inhibiting RASA1, thus promoting CRC cell growth [29]. These results indicate the potential of miR-31 as a biomarker and therapeutic target in CRC.

PTEN is also a target gene of miR-92a, and the expression of miR-92a is significantly upregulated in CRC patients with lymph node metastasis. Moreover, miR-92a induces epithelial to mesenchymal transition (EMT) and regulates cell growth, migration, and invasion by downregulating PTEN expression [30]. Analysis of clinicopathologic features showed that miR-92a overexpression is closely associated with advanced clinical stage, lymph node metastasis, distant metastasis, and poor overall survival (OS), which indicated that miR-92a could serve as a prognostic marker for CRC [31].

Several important oncogenic miRNAs are associated with the development and progression of CRC, including miR-29a [32], miR-32 [33], miR-95 [34], miR-96 [35], miR-103 [36], miR-181a [37,38], miR-182 [39], miR-196b [40], and miR-223 [41] (Table 1).

**Table 1:** Oncogenic miRNAs in CRC.

Names	Expression	Targets	Biological Events	References
miR-18a	↑	ATM	cell apoptosis, repair of DNA damage, sensitive to genotoxin (etoposide)	[21]
miR-21	↑	PTEN, PDCD4	cell proliferation, prognosis, response to adjuvant chemotherapy	[23,24,25,26]
miR-29a	↑	KLF4	cell invasion, metastasis, prognosis	[32]
miR-31	↑	FIH-1, RhoBTB1, RASA1	cell proliferation, migration, invasion, tumor growth, prognosis	[27,28,29]
miR-32	↑	PTEN	cell proliferation, migration, invasion, apoptosis	[33]
miR-92a	↑	PTEN	cell proliferation, migration, invasion, clinical stage, lymph node metastases, distant metastases, prognosis	[30,31]
miR-95	↑	SNX1	cell proliferation, tumor growth	[34]
miR-96	↑	TP53INP1, FOXO1, FOXO3a	cell proliferation	[35]
miR-103	↑	DICER, PTEN	cell proliferation, migration, tumor growth	[36]
miR-181a	↑	WIF-1, PTEN	cell proliferation, migration, invasion, tumor growth, liver metastasis, metabolic shift, EMT, advanced stage, distant metastasis, prognosis	[37,38]
miR-182	↑	-	tumor size, lymph node metastasis, TNM stage, prognosis	[39]
miR-196b	↑	FAS	cell apoptosis	[40]
miR-223	↑	-	cell proliferation, migration, invasion, clinical stage	[41]

**↑**: upregulated; -: unknown.

#### 2.1.2. Tumor Suppressor miRNAs

Certain miRNAs, such as miR-18a, are known as oncogenes in CRC, whereas they simultaneously possess the characteristics of tumor suppressors. miR-18a repression was shown to significantly increase cell proliferation and promote the anchorage-independent growth of colon adenocarcinoma HT-29 cells. Moreover, the expression of miR-18a and Kirsten-Ras (K-Ras) were inversely correlated in A431, HT-29, and WRL-68 cells, indicating that miR-18a specifically targets K-Ras but not other Ras isoforms [42].

The importance of miR-133a in CRC has been demonstrated [43]. The expression of miR-133a was significantly downregulated in 17 pairs of CRC tissues and three cell lines, including HT-29, SW-480, and SW-620. Fascin1 (FSCN1) was shown to be a direct target gene of miR-133a by dual luciferase reporter assay, and the protein expression of FSCN1 was negatively associated with that of miR-133a. Meanwhile, miR-133a could suppress cell invasion through direct inhibition of FSCN1. miR-133a was also shown to inhibit cell proliferation, migration, and overexpression of miR-133a-suppressed tumor growth and intrahepatic and pulmonary metastasis *in vivo*. Further investigation into the mechanisms of action of miR-133a showed that miR-133a acts via the MAPK signaling pathway by inhibiting the phosphorylation of ERK and MEK [44]. miR-133b can function as a tumor suppressor and negatively regulate TATA box-binding protein-like protein 1 (TBPL1), a novel target of miR-133b in CRC that is associated with cell proliferation [45]. In addition, the overexpression of miR-133b suppressed CRC cell invasion and migration and promoted apoptosis partly through the negative regulation of CXC chemokine receptor 4 (CXCR4) [46].

Investigation of the biological mechanisms and clinical significance of miR-194 in CRC showed that it is downregulated in tissues and cell lines. miR-194 inhibits CRC cell proliferation, apoptosis, migration, and invasion and is associated with tumor size, lymph node metastasis, and poor survival. miR-194 acts as a tumor suppressor in CRC by targeting the PDK1/AKT2/XIAP signaling pathway, and miR-194 could serve as a significant diagnostic and prognostic biomarker for CRC [47]. Downregulation of miR-194 was also associated with tumor size, differentiation, and TNM stage in CRC tissues. Furthermore, miR-194 promotes cell cycle arrest at the G1 phase. MAP4K4 was identified as direct target gene of miR-194 and shown to regulate MDM2 expression through the transcription factor c-Jun. These results indicate that miR-194 acts as a tumor suppressor by regulating the MAP4K4/c-Jun/MDM2 signaling pathway [48].

miR-126 was previously reported to play important roles in promoting vascular integrity and angiogenesis by vascular endothelial growth factor (VEGF) [49]. However, recent studies showed that miR-126 plays a role in tumor progression in CRC [50,51,52,53]. The expression of miR-126 is downregulated in primary CRC tissues and cell lines, and overexpression of miR-126 inhibits cell proliferation, migration, and invasion, and causes cell cycle arrest at the G0/G1 phase, though it is not associated with apoptosis. The knockdown of miR-126 promotes AKT and ERK1/2 activation by upregulating the expression of IRS-1, a target gene of miR-126 [51]. VEGF was identified as a direct target of miR-126, and DNA methylation-induced silencing of miR-126 partly contributes to tumor invasion and angiogenesis in CRC via the upregulation of VEGF [52]. miR-126 may also be a tumor suppressor by regulating CXCR4 through the AKT and ERK1/2 signaling pathways [53]. In brief, miR-126 may play an important role in CRC and could serve as an important biomarker.

**Figure 3:**
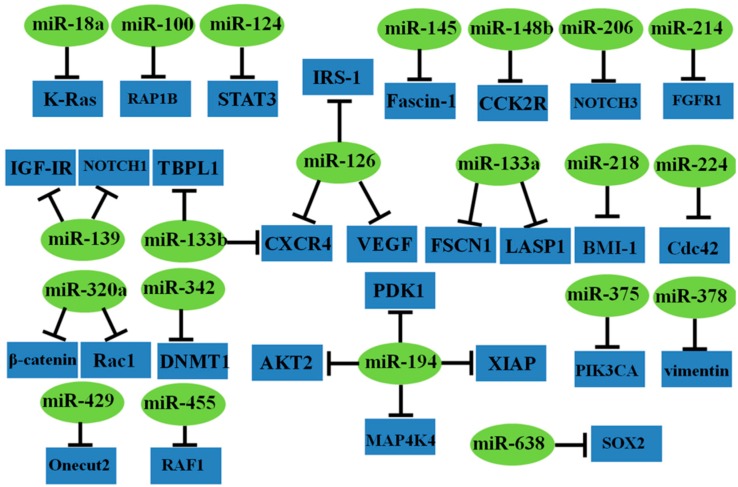
Representative tumor suppressor miRNAs and their target genes in CRC.

In addition to these common miRNAs, several other miRNAs are associated with CRC and could function as tumor suppressors, including miR-100 [54], miR-124 [55,56], miR-139 [57,58], miR-145 [59], miR-148b [60], miR-206 [61], miR-214 [62], miR-218 [63], miR-224 [64], miR-320a [65,66], miR-342 [67], miR-375 [68], miR-378 [69], miR-429 [70], miR-455 [71], and miR-638 [72] (Table 2, Figure 3). Several recent reviews have also systematically summarized these “small molecules with big functions” [73].

**Table 2:** Tumor suppressive miRNAs in CRC.

Names	Expression	Targets	Biological Events	References
miR-18a	-	K-Ras	cell proliferation, anchorage-independent growth	[42]
miR-100	↓	RAP1B	cell proliferation, invasion, apoptosis	[54]
miR-124	↓	STAT3	cell proliferation, apoptosis, tumor growth, differentiation, prognosis	[55,56]
miR-126	↓	IRS-1, VEGF, CXCR4	cells proliferation, migration, invasion, cell cycle arrest, angiogenesis, diagnosis	[50,51,52,53]
miR-133a	↓	FSCN1, LASP1	cell proliferation, invasion, migration, tumor growth, intrahepatic and pulmonary metastasis, phosphorylation of ERK/MEK	[43,44]
miR-133b	↓	TBPL1, CXCR4	cell proliferation, invasion, migration, apoptosis	[45,46]
miR-139	↓	IGF-IR, NOTCH1	cell proliferation, migration, invasion, apoptosis, tumor growth, cell cycle arrest	[57,58]
miR-145	↓	Fascin-1	cell proliferation, invasion, tumor growth, pulmonary metastasis	[59]
miR-148b	↓	CCK2R	cell proliferation, tumor growth, tumor size	[60]
miR-194	↓	PDK1, AKT2, XIAP, MAP4K4	cell proliferation, apoptosis, migration, invasion, cell cycle arrest, tumor growth, tumor size, differentiation, TNM stage, lymph node metastasis, prognosis	[47,48]
miR-206	↓	NOTCH3	cell proliferation, migration, apoptosis, cell cycle arrest	[61]
miR-214	↓	FGFR1	cell proliferation, migration, invasion, tumor growth, liver metastasis	[62]
miR-218	↓	BMI-1	cell proliferation, apoptosis, cell cycle arrest	[63]
miR-224	↓	Cdc42	cell migration	[64]
miR-320a	↓	β-catenin, Rac1	cell proliferation, migration, invasion, cell cycle arrest	[65,66]
miR-342	↓	DNMT1	cell proliferation, invasion, cell cycle arrest, tumor growth, lung metastasis	[67]
miR-375	↓	PIK3CA	cell proliferation, cell cycle arrest, tumor growth	[68]
miR-378	↓	vimentin	cell proliferation, invasion, tumor growth, tumor size, lymph node metastasis, clinical stage, prognosis	[69]
miR-429	↓	Onecut2	cell migration, invasion, EMT	[70]
miR-455	↓	RAF1	cell proliferation, invasion	[71]
miR-638	↓	SOX2	cell invasion, migration, EMT	[72]

**↓**: downregulated; -: unknown.

### 2.2. MicroRNAs in Clinical Applications

#### 2.2.1. miRNAs as Potential Biomarkers for CRC

Although current screening methods for CRC such as colonoscopy and the fecal occult blood test are commonly used, they are limited by their low specificity and sensitivity, as well as by high invasiveness, high cost, and increased risk [74]. Therefore, effective and stable methods to assess CRC are necessary. A growing number of studies have reported that miRNAs could function as potential diagnostic and prognostic biomarkers for CRC in stool, serum, plasma, and tissues as non-invasive methods.

miRNAs in stool: Certain miRNAs show higher expression levels in stool samples from CRC patients than in those from healthy controls. Ahmed *et al.* [75] identified 12 miRNAs (miR-7, -17, -20a, -21, -92a, -96, -106a, -134, -183, -196a, -199a-3p, and -214) that are upregulated in the stool of CRC patients. By contrast, eight miRNAs (miR-9, -29b, -127-5p, -138, -143, -146a, -222, and -938) were shown to be downregulated by PCR analysis.

miR-221 and miR-18a are significantly upregulated in CRC tissues compared with adjacent normal tissues. These two miRNAs showed increased expression levels in 198 stool samples from patients with CRC stages I and II and stages III and IV; however, there was no obvious association between their levels and the location of CRC, such as the proximal colon, the distal colon, and the rectum. Moreover, miR-221 and miR-18a expression levels are not associated with antibiotic intake [76]. However, these results have not been replicated and the small sample sizes of advanced adenoma and antibiotic intake groups may have caused bias so that larger-scale validation will be needed for further confirmation.

A study of fecal samples from 17 CRC patients and 28 healthy subjects identified several potential miRNA markers, and the expression of miR-223 and miR-451 was higher in CRC patients than in healthy controls. Fecal miR-223 showed a sensitivity of 76.5% and specificity of 96.4%, whereas fecal miR-451 produced a sensitivity of 88.2% and specificity of 100.0% [77]. Similarly, CRC patients had higher stool levels of miR-21 and miR-92a, but their expression was significantly downregulated after the removal of tumors, suggesting that they could be useful for CRC screening [78]. The immunochemical fecal occult blood test (iFOBT) has been widely used for CRC screening. However, a novel method termed the fecal microRNA test (FmiRT) is currently used to detect CRC. One study reported that among 117 CRC patients and 107 healthy volunteers, the expression of fecal miR-106a was significantly higher in iFOBT+ and iFOBT− patients than in healthy controls. Most importantly, 25% of CRC patients with false-negative iFOBT are likely to be true positive with the combined analysis of iFOBT and fecal miR-106a, which reveals the value of miR-106a to accurately diagnose CRC in cases of negative iFOBT results [79]. miR-135b could also be used as a potential non-invasive biomarker for CRC screening, although the expression of miR-135b is significantly downregulated after tumor removal, and there is no obvious relationship between the levels of miR-135b and proximal, distal, and colorectal lesions [80]. miR-144 is overexpressed both in the tissues and feces of CRC patients, with a sensitivity of 74% and a specificity of 87%, suggesting that it could be a diagnostic marker for CRC [81].

The abnormal DNA methylation of miR-34a in the stool is useful for the detection of CRC, as 63 of 82 samples were methylated, whereas only 2 of 40 healthy samples were methylated. Furthermore, the abnormal methylation of miR-34a was found to be correlated with lymph metastasis. Regarding miR-34b/c methylation, it was found in 74 of 79 cancer stool samples. These results indicate that methylation of miR-34a and miR-34b/c might play a role in the non-invasive screening and diagnosis of CRC [82].

Certain miRNAs, such as miR-143 and miR-145, show lower expression in the stool of CRC patients than in that of healthy controls [83]. The fecal expression levels of miR-4487 and miR-1295b-3p are significantly decreased in CRC patients with early stage I and II compared with that of normal controls [84]. These miRNAs in the stool could function as potential non-invasive molecular biomarkers for CRC diagnosis. However, additional studies are needed to further explore the role of these miRNAs in the detection of CRC (Table 3).

**Table 3:** miRNAs as potential biomarkers in stool of CRC.

Names	Sample Quantity	Expression	Sensitivity	Specificity	Biomarkers	References
miR-221	595	↑	62%	74%	diagnosis	[76]
miR-18a	595	↑	61%	69%	diagnosis	[76]
miR-223	45	↑	76.5%	96.4%	diagnosis	[77]
miR-451	45	↑	88.2%	100%	diagnosis	[77]
miR-21	246	↑	55.7%	73.3%	diagnosis	[78]
miR-92a	246	↑	71.6%	73.3%	diagnosis	[78]
miR-106a	224	↑	34.2%	97.2%	diagnosis	[79]
miR-135b	424	↑	78%	68%	diagnosis	[80]
miR-144	75	↑	74%	87%	diagnosis	[81]
miR-143	51	↓	-	-	diagnosis	[83]
miR-145	51	↓	-	-	diagnosis	[83]
miR-4478	56	↓	-	-	diagnosis	[84]
miR-1295b-3p	56	↓	-	-	diagnosis	[84]

**↑**: upregulated; **↓**: downregulated; -: unknown.

miRNAs in serum or plasma: Circulating miRNAs, which exist in the serum or plasma of CRC patients, can be important biomarkers for CRC screening and prognostic evaluation. Nine miRNAs (miR-18a, -20a, -21, -29a, -92a, -106b, -133a, -143, and -145) are differentially expressed in CRC patients [85]. Analysis of the association between the expression of miRNAs and clinicopathological features showed that the expression of miR-181b was associated with lymph node metastasis; however, none of the miRNAs was associated with age, tumor site, or tumor stage. Meanwhile, miR-181b showed a higher expression in the plasma of late stage CRC patients than in that of early stage patients. The plasma levels of miR-221 were increased in CRC and could play important roles in molecular diagnosis, with a sensitivity of 86% and a specificity of 41%, in addition to the value of miR-221 as a prognostic marker for differentiating CRC patients from controls. Circulating miR-221 levels are also correlated with p53 expression levels [86]. miR-21 was previously shown to negatively regulate tumor suppressor genes, and the levels of serum miR-21 were significantly increased in CRC in several studies. In a study analyzing 282 serum samples, including those from patients with CRC, adenomatous polyps, and normal controls, high expression of miR-21 in serum was significantly associated with tumor size, distant metastasis, and poor survival, suggesting that serum miR-21 levels could serve as a marker for the early diagnosis and prognosis of CRC [87]. The increased expression of serum miR-21 was also associated with clinical stage in CRC patients [88]. In addition, the expression of serum miR-21 was shown to be correlated with recurrence and mortality in CRC patients [89]. miR-183 was shown to serve as a circulating biomarker that is significantly overexpressed in plasma samples from CRC patients. Its expression decreased significantly after surgery, and the area under the receiver operating characteristic (ROC) curve for miR-183 was 0.829, which was higher than that of carcinoembryonic antigen (CEA) and carbohydrate antigen 19-9 (CA19-9). Furthermore, this study showed that miR-183 was associated with lymph node metastasis, distant metastasis, high TNM stage (III–IV), tumor recurrence, and poor OS [90]. Regarding miR-106a, there is no correlation between preoperative plasma expression and clinicopathological features such as lymph node metastasis and TNM stage, despite the fact that plasma miR-106a levels were significantly higher in CRC patients and decreased after surgery [91]. Studies have confirmed that miR-19a is dysregulated in FOLFOX chemotherapy-resistant patients, indicating that serum miR-19a could be a novel biomarker for predicting and monitoring resistance to FOLFOX in advanced CRC patients [92]. Serum expression of miR-18a and miR-29a is significantly upregulated in stage III CRC patients, and these findings indicated that both serum miR-18a and miR-29a levels could serve as promising markers for the identification of CRC patients [93]. These circulating miRNAs could be used as potential non-invasive diagnostic or prognostic molecular markers in CRC (Table 4).

Blood-based miRNAs appear to be more sensitive but less specific than fecal miRNAs for the diagnosis of CRC, and assays based on multiple miRNAs are more accurate for diagnosis than single-miRNA assays. In addition, miRNA-based diagnoses appear to be more accurate in Asian than in Caucasian patients [94].

**Table 4:** miRNAs as potential biomarkers in blood and tissues of CRC.

Names	Samples	Sample Quantity	Expression	Sensitivity	Specificity	Biomarkers	References
miR-221	plasma	140	↑	86%	41%	diagnosis and prognosis	[86]
miR-21	serum	282	↑	82.8%	90.6%	diagnosis and prognosis	[87]
miR-106a	plasma	97	↑	62.3%	68.2%	diagnosis	[91]
miR-19a	serum	72	↑	66.7%	63.9%	predicting and monitoring resistance to FOLFOX	[92]
miR-18a	serum	56	↑	-	-	diagnosis	[93]
miR-29a	serum	56	↑	-	-	diagnosis	[93]
miR-183	plasma	179	↑	73.7%	88.5%	tumor recurrence, diagnosis and prognosis	[90]
miR-126	tissues	92	↓	-	-	prognosis	[95]
miR-630	tissues	206	↑	-	-	prognosis	[96]
miR-378	tissues	84	↓	-	-	prognosis	[69]

**↑**: upregulated; **↓**: downregulated; -: unknown.

miRNAs in tissues: miRNAs in tissues could also be developed as prognostic biomarkers. miR-126 is downregulated in CRC tissues and associated with distant metastasis, TNM stage, and poor survival, indicating its clinical significance as a prognostic biomarker in CRC [95]. RT-PCR analyses in 206 CRC patients showed that miR-630 is upregulated in tumor tissues compared with adjacent normal tissues, and its expression is associated with tumor invasion, lymph node metastasis, distant metastasis, and TNM stage [96]. miR-378 was shown to be downregulated in 84 paired tissues and inhibited cell growth as well as invasion, acting as a tumor suppressor and independent prognostic factor in CRC [69]. miR-92a was suggested as a novel potential biomarker for the diagnosis of CRC in a meta-analysis that included six studies with a total of 521 CRC patients and 379 normal controls [97] (Table 4).

It is worth noting that the replication of these studies and the statistical power of correlations may be important influencing factors for the reliability of the studies. Indeed, most of these results have not been replicated. Moreover, statistical power would be influenced by either the small number of sample size such as miR-223, miR-451, miR-34a or the large number of miRNAs being sampled. Another noticeable problem is that some of the reported miRNAs in Table 3 and Table 4 that putatively serve as biomarkers are likely false positives as they were not found in many of these studies. Thus, the above results need to be identified in multiple studies with larger scale validation across multiple centers and different populations for further confirmation.

#### 2.2.2. miRNAs in the Evaluation of Treatment Response in CRC Patients

Surgical resection is currently the main treatment for CRC, with adjuvant chemotherapy and radiotherapy used concomitantly. In CRC patients at advanced stages, clinical practice has demonstrated that palliative chemotherapy, such as the use of fluorouracil (5-FU) combined with irinotecan and oxaliplatin, can successfully improve survival. Chemotherapy resistance in CRC has become a severe problem and an important challenge that needs to be overcome.

miRNAs in chemotherapy: In a cohort of 295 patients, miR-17-5p was found to be upregulated in chemoresistant patients compared with chemosensitive patients. Moreover, high expression of miR-17-5p in CRC patients is associated with worse prognosis, distant metastases, and higher clinical stage, indicating that miR-17-5p could function as a predictive biomarker for chemotherapy response [98]. In a search for potential and novel molecular biomarkers to predict the resistance to FOLFOX, which is the most commonly used first-line therapy for advanced CRC patients, 62 miRNAs were found to be differentially expressed, with a fold-change >2 by microarray analysis. Among these miRNAs, miR-19a was significantly upregulated in the serum of drug-resistant patients, and it could discriminate resistant patients from responsive ones with sensitivity of 66.7% and specificity of 63.9%. These results suggested that serum miR-19a could act as a potential biomarker for resistance to FOLFOX chemotherapy regimens in advanced CRC patients [92].

Bevacizumab is a widely accepted and used monoclonal antibody and it has been shown to improve clinical results; however, it is associated with several adverse events. Two circulating miRNAs (miR-1254 and miR-579) have been shown to be upregulated in CRC patients with bevacizumab-induced cardiotoxicity and could help distinguish between bevacizumab-induced cardiotoxicity and acute myocardial infarction (AMI). Therefore, these two miRNAs could be developed as biomarkers for the clinical diagnosis of bevacizumab-induced cardiotoxicity [99]. The expression levels of miR-200 family members, including miR-200a, -200b, -200c, -141, and miR-429, were shown to be higher in the tumor tissues of 127 stage I–III CRC patients. In addition, patients showing high levels of miR-200a, miR-200c, miR-141, or miR-429 that were treated with fluoropyrimidines had better overall and disease-free survival (DFS). Studies have shown that overexpression of miR-429 combined with 5-FU treatment inhibits cell invasion in the LoVo cell line. These results indicate that these miRNAs may possess the capacity to identify CRC patients who would benefit from adjuvant chemotherapy [100]. Zhang *et al.* [101] reported that a novel p53/miR-520g/p21 signaling pathway plays an important role in the response of CRC patients to chemotherapy. miR-520g was shown to mediate drug resistance to 5-FU or oxaliplatin-induced apoptosis *in vitro* and to suppress the ability of 5-FU to inhibit tumor growth *in vivo* by downregulating p21 expression. Meanwhile, p53 downregulated miR-520g expression, and the inhibition of miR-520g in p53 (−/−) cells increased the sensitivity to 5-FU treatment.

miRNAs in radiotherapy: Resistance to radiation has been shown to be one of the most severe challenges in CRC treatment. Several miRNAs may be involved in the biological response to radiation therapy.

In one study, miR-124 was shown to be significantly downregulated in CRC tissues and cell lines, and miR-124 sensitized CRC cells to ionizing radiation both *in vitro* and *in vivo*. Furthermore, this study demonstrated that PRRX1, a new EMT inducer, could be a novel target gene of miR-124, and knockdown of PRRX1 sensitized CRC cells to radiation [102]. After exposure to different doses of X-rays (0, 2, 4, 6, and 8 Gy), all of the RNAs and proteins in CRC cell lines were extracted 24 h after irradiation, which showed that the expression of miR-221 was increased with the increase in the irradiation dose, whereas PTEN protein expression levels were reduced. These results showed that anti-miR-221 could improve the radiosensitivity of CRC cells by upregulating PTEN, indicating that miR-221 may act as a potential useful biomarker and therapeutic target in CRC [103].

## 3. Small Interfering RNAs in CRC

SiRNAs are sncRNAs of 21–25 nt that are formed by Dicer of the RNAase III family, which is a specific enzyme for double-stranded RNAs. They are generally not endogenously produced, but function as significant members of siRISCs and are used to silence target mRNAs by exogenously introduced into cells by the researchers and to. The following siRNAs were recently found to be “star ncRNAs” in CRC.

Stromal interaction molecule 1 (STIM1) is a factor associated with cancer progression that is upregulated in highly invasive CRC cell lines and tissues, and promotes cell metastasis both *in vitro* and *in vivo*. SiRNA-mediated silencing of STIM1 showed the opposite effect. Moreover, silencing of STIM1 inhibited EMT and STIM1 was shown to be a direct target of miR-185 [104]. Leukemia inhibitory factor (LIF) expression was shown to be stimulated by hypoxia in human CRC cells, and LIF induction under hypoxia was mainly mediated by HIF-2α. The knockdown of endogenous HIF-2α by siRNA largely inhibited the induction of LIF by hypoxia [105]. The peroxiredoxins (Prdxs), as important scavengers of reactive oxygen species (ROS), are upregulated in CRC tissues and cell lines. SiRNA-mediated silencing of the Prdx2 gene decreases cell proliferation, increases cell apoptosis, and induces endogenous production of ROS. Furthermore, Prdx2 was shown to be involved in the regulation of the Wnt/β-catenin signaling pathway [106].

## 4. Piwi-Interacting RNAs in CRC

PiRNAs are a novel type of sncRNAs of 26–31 nt that specifically interact with the P-element-induced wimpy testis (Piwi) protein. They can control and silence the transposable elements (TEs) to protect the genome [107,108], whereas the uncontrolled expression of TEs can lead to the loss of genome integrity.

Recent studies showed that they play crucial roles in carcinogenesis in humans [109,110]. PiR-651 was found to be significantly upregulated in gastric cancer and colon cancer tissues [111]. One study showed that piRNAs are important determinants of CRC risk by binding to Piwi, and that genetic variants in piRNAs modulate CRC susceptibility. They identified seven piRNA SNPs in a search of all known human piRNAs available, and investigated the association between these seven SNPs and the risk of CRC [112]. The SNP rs11776042 in piR-015551, which may be generated from a type of lncRNA called LNC00964-3, was significantly associated with a decreased risk of CRC in an additive model; however, the protective effect was not significant after multiple comparison correction. Nevertheless, the results indicated that piR-015551 may be involved in the development of CRC.

## 5. Long Non-Coding RNAs in CRC

LncRNAs are newly identified ncRNAs that have been shown to play key regulatory roles in many diseases including cancer, and have emerged as significant participants in tumor development and progression. Additionally, the close association between dysregulated lncRNAs and the diagnosis and prognosis of CRC patients indicated their potential capacity as novel biomarkers or therapeutic targets. Several recent reports and reviews have described the role of lncRNAs in cancer, including CRC [8,113,114,115,116]. Furthermore, with the development of high-throughput microarray assays and bioinformatic approaches, many differently expressed lncRNAs have been identified in CRC. In a microarray expression study, 23,920 lncRNAs were identified in paired CRC tissues, with 762 remarkably differentially expressed lncRNAs [117]. A different study [118] focused on screening lymph node metastasis-associated lncRNAs in CRC patients, resulting in the identification of 1133 lncRNAs differentially expressed in metastatic lymph nodes (MLNs) compared with normal lymph nodes (NLNs), of which 260 were upregulated and 873 were downregulated. Here, we summarized several representative lncRNAs that are commonly detected in CRC.

### 5.1. MALAT1

Metastasis-associated lung adenocarcinoma transcript 1 (MALAT1) is a functional lncRNA that is located on chromosome 11q13 and was reported to be involved in multiple cancers including osteosarcoma [119], hepatocellular carcinoma [120], and esophageal squamous cell carcinoma [121]. Its biological functions in the development and progression of CRC have recently attracted attention.

In a study of 146 CRC tissue samples obtained from stage II and III patients, the expression of MALAT1 was 2.26 times higher than that in adjacent normal tissues and significantly overexpressed in male patients. However, there was no association between MALAT1 and other clinicopathological factors. Moreover, patients with higher MALAT1 expression had shorter DFS and OS. The above results indicate that the overexpression of MALAT1 could act as a negative prognostic marker for CRC patients with stage II/III diagnoses [122]. At the same time, the expression of MALAT1 was upregulated in LoVo and SW620 cells compared with SW480, HCT116, LS174T, and HCT8 cells, and was higher in CRC tissues [123]. MALAT1 significantly increased colony formation and migration of cells in soft agar colony formation and wound healing assays *in vitro* and it promoted tumor growth and lung metastasis in a mouse model *in vivo*. Regarding the underlying molecular mechanisms, MALAT1 showed the ability to competitively bind to the tumor suppressor gene SFPQ and release the proto-oncogene PTBP2 from the SFPQ/PTBP2 complex, while the increased SFPQ-detached PTBP2 could promote tumor growth and migration. At the same time, the 3′ end of MALAT1 could play crucial roles in promoting the invasion and migration of CRC cells [124]. Meanwhile, MALAT1 is upregulated in CRC tissues and its expression is higher in tissues with lymph node metastasis, as shown by Yang and colleagues [125]. The overexpression of MALAT1 promoted CRC cell proliferation, invasion, and migration *in vitro* and tumor growth and metastasis *in vivo*. Interestingly, MALAT1 might promote these biological functions by targeting PRKA kinase anchor protein 9 (AKAP-9), although further investigation is necessary.

**Figure 4:**
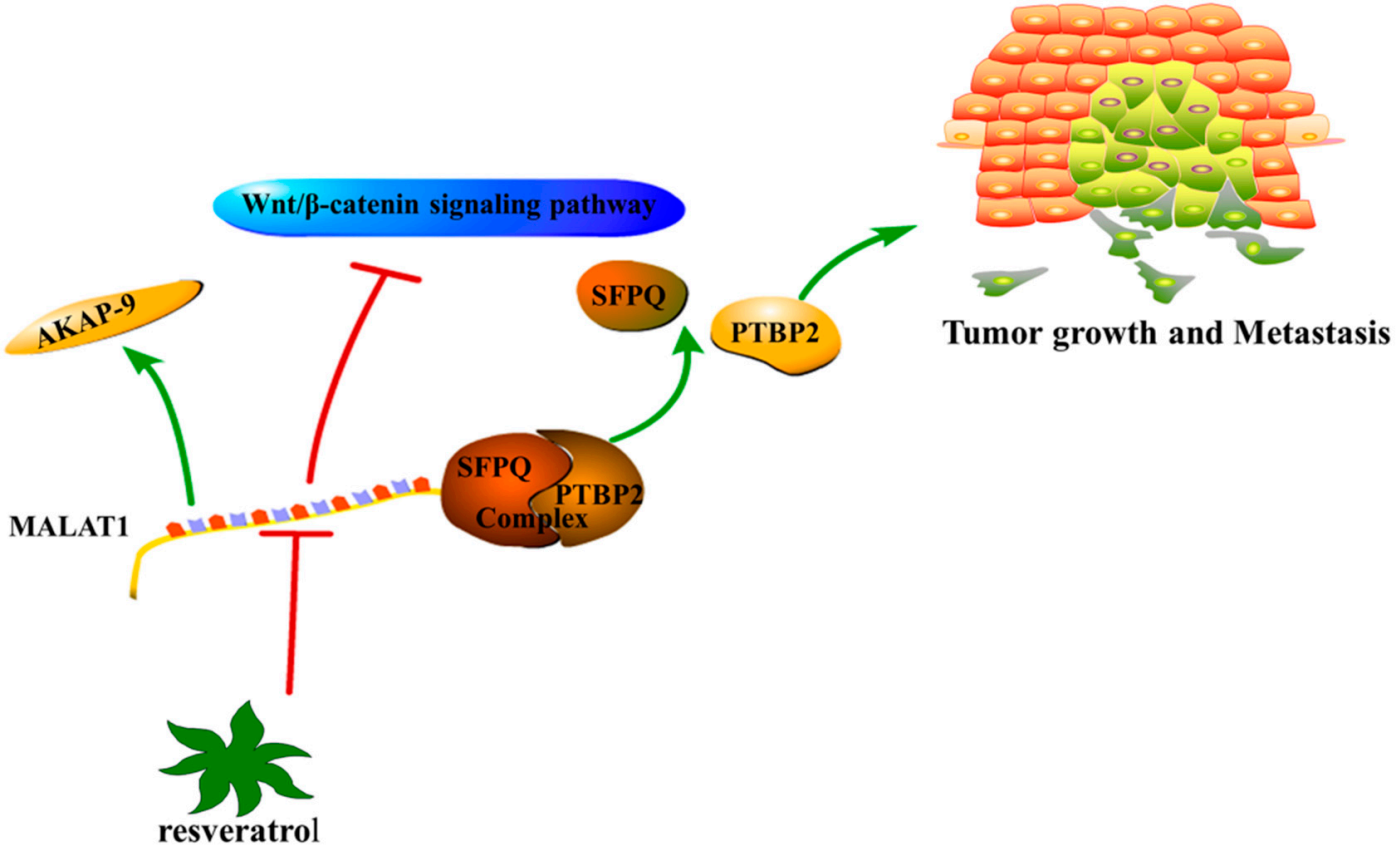
The biological mechanisms of MALAT1 in CRC. Resveratrol could suppress CRC cell invasion and migration by inhibiting Wnt/β-catenin signaling pathway through downregulating MALAT1; MALAT1 might promote CRC development via targeting AKAP-9; MALAT1 could bind to SFPQ and release PTBP2 from the SFPQ/PTBP2 complex that promotes tumor growth and migration.

Resveratrol, which is extracted from the Chinese herbal medicine “polygonum cuspidatum”, has been suggested as a new Chinese medicine monomer anticancer drug for different cancers [126,127]. Furthermore, it has been shown to be effective against CRC through the caspase/cyclin-CDK pathways [128] and the TGF-β1/Smads signaling pathway [129]. It significantly inhibited the expression of MALAT1 in LoVo cells by directly inhibiting the promoter activity of MALAT1, and it inhibited cell proliferation, invasion, and migration in a dose-dependent manner in CRC. The expression of MALAT1 was statistically significantly associated with clinicopathological features including metastasis and invasion. MALAT1 was shown to increase the cytoplasmic levels of β-catenin, concomitant with the downregulation of its nuclear expression. The knockdown of MALAT1 could downregulate the expression of β-catenin downstream target genes such as c-myc and MMP-7. Resveratrol suppressed CRC cell invasion and migration by inhibiting the Wnt/β-catenin signaling pathway through the downregulation of MALAT1 [130] (Figure 4).

### 5.2. HOTAIR

HOX transcript antisense intergenic RNA (HOTAIR) was found to transcribe the homeobox *C* gene on chromosome 12q13.13 in an antisense manner. An increasing number of studies have shown that the expression of HOTAIR is closely associated with a variety of cancers, suggesting that it is a cancer-associated lncRNA [131,132]. Moreover, relevant studies have demonstrated that HOTAIR could function as an underlying biomarker for lymph node metastasis, prognosis, and a therapeutic target in tumors [133,134,135,136].

Several recent studies have analyzed the role of HOTAIR in CRC. The expression of HOTAIR was found to be upregulated in cancer tissues from 95 CRC patients. The investigation of the association between HOTAIR tag SNPs and the risk of CRC showed that patients with the rs7958904 CC genotype had a decreased risk of CRC and the rs7958904 C allele was associated with the inhibition of cell proliferation compared with the rs7958904 G allele, indicating that genetic variants of HOTAIR play important roles in CRC [137]. Other studies showed that high HOTAIR expression is accompanied with less differentiated histology, greater tumor depth, and liver metastasis. Patients with high HOTAIR expression had poor prognosis, indicating that HOTAIR could be an independent prognostic indicator in CRC patients by multivariate analysis. HOTAIR also promotes cell invasion in CRC and shows a close correlation with members of polycomb-repressive complex 2 (PRC2), such as SUZ12, EZH2, and H3K27me3 in a cDNA array [138]. Similarly, high expression of HOTAIR is correlated with tumor depth, lymph node metastasis, organ metastasis, histological differentiation, vascular invasion, and advanced tumor stage, and patients with HOTAIR upregulation show a poor clinical prognosis such as lower metastasis-free survival and OS. Moreover, HOTAIR significantly promotes cell migration and invasion, and has a mild effect on the inhibition of cell proliferation. The knockdown of HOTAIR upregulated E-cadherin and downregulated vimentin and matrix metalloproteinase 9 (MMP9), indicating that HOTAIR might be an important factor in the process of EMT [139]. In addition to CRC tissues, the expression of HOTAIR in patients’ blood was higher than that in healthy controls. The upregulation of HOTAIR in primary tumors and blood were both associated with poor prognosis, which suggested that the expression of HOTAIR in blood could also function as a prognostic marker in CRC [140].

### 5.3. H19

H19 is a maternally imprinted oncofetal ncRNA that is located on chromosome 11p15.5. H19 is highly expressed during the early stages of embryogenesis, whereas it is downregulated after birth, without the function of a coding protein [141]. Recent studies showed that the dysregulation of H19 plays a role in different cancers including CRC. In a study conducted in 2002, the H19 differentially methylated region (DMR) as well as a DMR upstream of exon 3 of IGF2 were shown to be hypo-methylated in both CRC tissues and normal mucosa [142], whereas a 2012 study showed that hypo-methylation of the sixth CTCF-binding site in the DMR of IGF2/H19 is related to loss of imprinting (LOI), indicating that the LOI of IGF2 plays an important role in CRC [143]. H19 was also strongly expressed in the liver metastasis of colon carcinoma via *in situ* hybridization (ISH) [144]. H19 could act as the precursor of miR-675, since both of H19 and miR-675 are upregulated in CRC tissues and cell lines, and the tumor suppressor retinoblastoma (RB) was shown to be a direct target gene of miR-675. Based on these results, the H19/miR-675/RB pathway was suggested to play crucial roles in CRC, and both H19 and miR-675 are important factors associated with CRC [145].

### 5.4. CCAT Family

Colon cancer-associated transcript 1 (CCAT1) maps to chromosome 8q24.21, which was first discovered in CRC with strong expression [146]. CCAT1 is upregulated both in the early phase of tumors, such as adenomatous polyps and tumor-proximal colonic epithelium, and in the later stages of CRC, such as liver or peritoneal metastasis. Moreover, CCAT1 is upregulated in the peripheral blood of CRC patients and in CRC-associated lymph nodes. CCAT1 expression has been investigated during the development of colon carcinoma: from normal tissues, adenoma, invasive carcinoma to lymph node and distant metastasis. The expression of CCAT1 was upregulated during the colon adenoma-carcinoma sequence and through all disease stages [147]. Furthermore, CCAT1-specific peptide nucleic acid (PNA)-based molecular beacons (TO-PNA-MB) were shown to generate a specific fluorescence signal when hybridized to the specific CCAT1 target in living cells and in human biopsies, which demonstrated that CCAT1 TO-PNA-MB could be a novel diagnostic tool for the detection of CRC [148]. CCAT1-L, as a novel CRC-specific lncRNA, was shown to be abundantly transcribed from a locus 515 kb upstream of MYC, and CCAT1-L could influence MYC transcriptional regulation and promote long-range chromatin looping in which CCAT1-L may interact with CTCF and modulate chromatin conformation [149].

Colon cancer-associated transcript 2 (CCAT2) was recently identified as a novel lncRNA in colon cancer in 2013, and it maps to the highly conserved 8q24.21 region encompassing the rs6983267 SNP, whose expression is not only upregulated in CRC tissues, but also in microsatellite-stable (MSS) cancers compared with microsatellite-unstable (MSI-H) cancers and normal mucosae. Moreover, CCAT2 was shown to promote cancer growth, metastasis, and induce chromosomal instability, and CCAT2 upregulated MYC expression through the activation of the Wnt signaling pathway by promoting TCF7L2 transcriptional activity rather than by increasing the quantity of TCF7L2 [150]. In addition to its role in CRC, CCAT2 is involved in breast cancer [151] and non-small cell lung cancer [152], indicating that it may play an important role in tumorigenesis.

### 5.5. Other Significant LncRNAs in CRC

Several lncRNAs have been shown to be involved in the development of CRC, including LOC285194 [153,154], uc.73 [155], uc.388 [155], lincRNA-p21 [156,157], GAS5 [158], ncRAN [159], ncRuPAR [160], MEG3 [161], RP11-462C24.1 [162], PRNCR1 [163], PVT-1 [164], CRNDE [165,166], HULC [167], PCAT-1 [168], BANCR [169,170], UCA1 [171], ATB [172], LINC01296 [173], and CCAL [174]. The associated biological processes and putative roles of these lncRNAs are described in Table 5.

However, their roles as diagnostic or prognostic biomarkers in CRC have not been identified in multiple studies, although some of these were identified in other cancers such as UCA1, BANCR, PCAT-1, and HULC, and these results need further confirmation.

**Table 5:** Other significant lncRNAs in CRC.

Names	Location	Expression	Biological Events	Putative Roles	References
LOC285194	Chr3q13.31	↓	tumor size, TNM stage, distant metastasis, prognosis, p53 transcription target, repression of miR-211, cell growth	diagnostic and prognostic biomarker	[153,154]
uc.73	Chr2q22.3	↓	overall survival	diagnostic and prognostic biomarker	[155]
uc.388	Chr12q13.13	↓	distal location of CRC	diagnostic and prognostic biomarker	[155]
lincRNA-p21	Chr6p21.2	↓	higher expression in rectum, stage III tumors, pT and vascular invasion, sensitivity of radiotherapy by targeting the Wnt/β-catenin signaling pathway, cell apoptosis, promote pro-apoptosis gene Noxa expression	sensitivity of CRC radiotherapy	[156,157]
GAS5	Chr1q25.1	↓	tumor size, histological grade, TNM stage, prognosis, cell proliferation	prognostic biomarker	[158]
ncRAN	Chr17q25.1	↓	histological grade, tumors with liver metastases, prognosis, cell migration, invasion	diagnostic and prognostic biomarker	[159]
ncRuPAR	Chr5q13.3	↓	lymph node metastasis, distant metastasis, Duck’s stage, histological grade, TNM stage, negatively associated with PAR-1	diagnostic biomarker	[160]
MEG3	Chr14q32.2	↓	histological grade, tumor invasion, TNM stage, prognosis, cell proliferation	diagnostic and prognostic biomarker	[161]
RP11-462C24.1	Chr4q25	↓	distant metastasis, prognosis	prognostic biomarker	[162]
PRNCR1	Chr8q24.21	-	SNPs in PRNCR1 may be involved in the risk of CRC (rs13252298, rs1456315, rs7007694, rs16901946 and rs1456315)	-	[163]
PVT-1	Chr8q24.21	↑	cell proliferation, invasion, apoptosis, prognosis	prognostic biomarker	[164]
CRNDE	Chr16q12.2	↑ (tissue and plasma)	regulating cellular metabolism by insulin/IGFs, a downstream target of the PI3K/Akt/mTOR pathway or Raf/MAPK pathway	diagnostic biomarker	[165,166]
HULC	Chr6p24.3	↑ (colorectal hepatic metastasis)	neither expressed in primary CRC samples nor normal tissues but upregulated in colorectal hepatic metastasis	-	[167]
PCAT-1	Chr8q24.21	↑	distant metastasis, prognosis	prognostic biomarker	[168]
BANCR	Chr9	↑ or ↓	lymph node metastasis, tumor stage, contribute to cell migration by inducing EMT via an MEK/ERK-dependent mechanism, cell proliferation, apoptosis, G0/G1 cell cycle arrest, targeting p21	therapeutic application	[169,170]
UCA1	Chr19p13.12	↑	cell proliferation, apoptosis, cell cycle, tumor size, histological grade, tumor depth, prognosis	diagnostic and prognostic biomarker	[171]
ATB	Chr14	-	tumor size, tumor depth, lymphatic invasion, vascular invasion, lymph node metastasis, hematogenous metastases, prognosis	prognostic biomarker	[172]
LINC01296	Chr14q11.2	↑	prognosis	prognostic biomarker	[173]
CCAL	-	↑	promoting CRC progression and induced multidrug resistance by targeting activator protein 2α, shorter overall survival, worse response to adjuvant chemotherapy	therapeutic application	[174]

**↑**: upregulated; **↓**: downregulated; -: unknown.

## 6. Small Nucleolar RNAs in CRC

SnoRNAs are a subclass of sncRNAs, ranging in size from 60 to 300 nt, that exist in the nucleolus of eukaryotic cells and are involved in the modification of ribosomal RNAs (rRNAs) [175]. SnoRNAs are located within introns of protein-coding genes and are transcribed by RNA polymerase II. SnoRNAs are classified as box C/D snoRNAs and box H/ACA snoRNAs, and box C/D snoRNAs are responsible for 2′-*O*-ribose methylation of rRNAs [176], whereas box H/ACA snoRNAs are responsible for pseudouridylation of rRNAs [177]. Several snoRNAs show differential expression patterns in many human diseases [178] including several malignancies such as breast cancer [179], prostate cancer [180], lung cancer [181], and hepatocellular carcinoma [182]. Their biological behaviors and functions in cancer have been summarized previously [183,184]. SnoRNAs could serve as reference genes to test the expression of miRNAs [185].

In the present review, we focused on current studies assessing the association of snoRNAs with carcinogenesis in CRC. One study [186] showed that GAS5-derived snoRNA expression was influenced by doxorubicin-induced DNA damage under the control of p53 in CRC cell lines and was not affected by Dicer. This study also found that p53 expression was correlated with GAS5-derived snoRNA levels in CRC. At the same time, a positive correlation was shown between the expression levels of miR-34a and both snoRNA U44 and snoRNA U47 in CRC samples. The host gene-associated 5′-CpG islands of snoRNAs such as SNORD123, U70C, and ACA59B were completely unmethylated in the normal colon mucosa, whereas they were hypermethylated in CRC cell lines, and these hypermethylations were closely involved in transcriptional silencing in CRC cells. However, the hypermethylation of snoRNAs is not limited to CRC, and it is a common phenomenon in other cancers, especially in leukemias, as shown in a DNA methylation microarray platform [187].

## 7. Small Nuclear RNAs in CRC

The snRNAs form the core components of the spliceosome and catalyze the removal of introns from pre-mRNA [188]. SnRNAs can form complexes with several proteins to form small nuclear ribonucleo-proteins (snRNPs). There are five major classes of snRNAS, including U1, U2, U4, U5, and U6. The expression of U2 snRNA fragments (RNU2-1f) was shown to be stable both in the serum and plasma of CRC patients, with a sensitivity of 97.7% and specificity of 90.6%, indicating that it could be a potential diagnostic biomarker for CRC. The RNU2-1f assay might correctly identify CRC patients as early as UICC stage II, suggesting that it could function as a potential non-invasive screening method for detecting early CRC with a good prognosis [189]. In addition to its use in CRC, RNU2-1f was also shown to be a diagnostic marker for cholangiocarcinoma [190].

## 8. Circular RNAs in CRC

Circular RNAs (circRNAs) are a special kind of endogenous ncRNA molecule, and they are the most popular RNAs in research recently. Animal genomes can express thousands of circRNAs from different genomic locations, and approximately 2000 human, 1900 mice, and 700 nematode circRNAs were identified using sequencing; however, the true number of circRNAs is thought to be higher [191]. CircRNAs play important roles in the regulation of gene expression at the transcriptional or post-transcriptional level. Certain circRNAs act as highly stable sponges for specific miRNAs, such as CiRS-7 and SRY for miR-7 and miR-138, and are involved in competing endogenous RNA networks [192]. Compared with the traditional linear RNAs, circRNAs form a closed loop structure generated by the head-to-tail splicing of exons without 5′ to 3′ polarity or a polyadenylated tail [193]. This unique structure may be attributed to the fact that exon circularization is dependent on flanking intronic complementary sequences [194]. They are not affected by RNA enzymes, resulting in their stable expression, and they are not easily degraded.

Recently, researchers showed that circRNAs are involved in the development of several types of diseases including cancer [195,196,197]. In CRC, there are 39 circRNAs differentially expressed in the normal colon mucosa and CRC samples, 11 of which are upregulated and 28 are downregulated [198]. The ratio of circular to linear RNA isoforms is lower in tumors than in normal samples, and it is even lower in CRC cell lines. Furthermore, this ratio was shown to be negatively correlated with the proliferation index. However, further studies are needed to explore their roles and mechanisms in CRC.

## 9. Transfer RNAs in CRC

Transfer RNA (tRNA) is an adaptor molecule composed of RNA, 76 to 90 nts in length [199]. tRNAs mainly carry amino acids into the ribosomes, and they are involved in protein synthesis under the guidance of mRNA. It is noteworthy that tRNAs could be cleaved into tRNA fragments (tRFs) when cells are exposed to various stresses with a potential impact on cell physiology such as translation, apoptosis, disease progression, and especially cancer [200]. For example, hypoxic stress can induce the production of tRFs, and they could suppress the development of breast cancer metastasis through binding to oncogenic RNA-binding protein YBX1 and displacing pro-oncogenic transcripts [201]. Although tRNAs have yet to be linked specifically to CRC, this may represent a potential future avenue of research.

## 10. Conclusions and Future Perspectives

NcRNAs, such as miRNAs, lncRNAs, siRNAs, snoRNAs, snRNAs, circRNAs, and piRNAs, have recently been shown to play a role in the development and progression of various cancers including CRC, and they have been identified as novel diagnostic and prognostic biomarkers. The growing number of studies on ncRNAs in CRC has increased our understanding of their molecular mechanisms and biological processes, which has opened new fields of research in CRC.

In the present review, we comprehensively summarized the findings of recent original studies and reviews on the role of ncRNAs in CRC, highlighting their usefulness and potential roles. Our findings suggest that these “star ncRNAs” will function as strong therapeutic tools in the clinical treatment of CRC in the future.

## References

[1] International Human Genome Sequencing Consortium (2004). Finishing the euchromatic sequence of the human genome. Nature.

[2] Palazzo A.F., Lee E.S. (2015). Non-coding RNA: What is functional and what is junk?. Front. Genet..

[3] Kaikkonen M.U., Lam M.T., Glass C.K. (2011). Non-coding RNAs as regulators of gene expression and epigenetics. Cardiovasc. Res..

[4] Li M., Izpisua Belmonte J.C. (2015). Roles for noncoding RNAs in cell-fate determination and regeneration. Nat. Struct. Mol. Boil..

[5] Piccoli M.T., Gupta S.K., Thum T. (2015). Noncoding RNAs as regulators of cardiomyocyte proliferation and death. J. Mol. Cell. Cardiol..

[6] Huang J., Peng J., Guo L. (2015). Non-Coding RNA: A new tool for the diagnosis, prognosis, and therapy of small cell lung cancer. J. Thorac. Oncol..

[7] Ma L., Bajic V.B., Zhang Z. (2013). On the classification of long non-coding RNAs. RNA Biol..

[8] Han D., Wang M., Ma N., Xu Y., Jiang Y., Gao X. (2015). Long noncoding RNAs: Novel players in colorectal cancer. Cancer Lett..

[9] Torre L.A., Bray F., Siegel R.L., Ferlay J., Lortet-Tieulent J., Jemal A. (2015). Global cancer statistics, 2012. CA Cancer J. Clin..

[10] Allemani C., Weir H.K., Carreira H., Harewood R., Spika D., Wang X.S., Bannon F., Ahn J.V., Johnson C.J., Bonaventure A. (2015). Global surveillance of cancer survival 1995–2009: Analysis of individual data for 25,676,887 patients from 279 population-based registries in 67 countries (CONCORD-2). Lancet.

[11] Wang J., Song Y.X., Wang Z.N. (2015). Non-coding RNAs in gastric cancer. Gene.

[12] Lee R.C., Feinbaum R.L., Ambros V. (1993). The *C. elegans* heterochronic gene lin-4 encodes small RNAs with antisense complementarity to lin-14. Cell.

[13] Amirkhah R., Schmitz U., Linnebacher M., Wolkenhauer O., Farazmand A. (2015). MicroRNA-mRNA interactions in colorectal cancer and their role in tumor progression. Genes Chromosomes Cancer.

[14] Tokarz P., Blasiak J. (2012). The role of microRNA in metastatic colorectal cancer and its significance in cancer prognosis and treatment. Acta Biochim. Pol..

[15] Hur K. (2015). MicroRNAs: Promising biomarkers for diagnosis and therapeutic targets in human colorectal cancer metastasis. BMB Rep..

[16] Hollis M., Nair K., Vyas A., Chaturvedi L.S., Gambhir S., Vyas D. (2015). MicroRNAs potential utility in colon cancer: Early detection, prognosis, and chemosensitivity. World J. Gastroenterol..

[17] Rossi S., di Narzo A.F., Mestdagh P., Jacobs B., Bosman F.T., Gustavsson B., Majoie B., Roth A., Vandesompele J., Rigoutsos I. (2012). MicroRNAs in colon cancer: A roadmap for discovery. FEBS Lett..

[18] Okugawa Y., Toiyama Y., Goel A. (2014). An update on microRNAs as colorectal cancer biomarkers: Where are we and what’s next?. Expert Rev. Mol. Diagn..

[19] Tao J., Wu D., Li P., Xu B., Lu Q., Zhang W. (2012). MicroRNA-18a, a member of the oncogenic miR-17-92 cluster, targets Dicer and suppresses cell proliferation in bladder cancer T24 cells. Mol. Med. Rep..

[20] Morimura R., Komatsu S., Ichikawa D., Takeshita H., Tsujiura M., Nagata H., Konishi H., Shiozaki A., Ikoma H., Okamoto K. (2011). Novel diagnostic value of circulating miR-18a in plasma of patients with pancreatic cancer. Br. J. Cancer.

[21] Wu C.W., Dong Y.J., Liang Q.Y., He X.Q., Ng S.S., Chan F.K., Sung J.J., Yu J. (2013). MicroRNA-18a attenuates DNA damage repair through suppressing the expression of ataxia telangiectasia mutated in colorectal cancer. PLoS ONE.

[22] Dong H., Strome S.E., Salomao D.R., Tamura H., Hirano F., Flies D.B., Roche P.C., Lu J., Zhu G., Tamada K. (2002). Tumor-associated B7-H1 promotes T-cell apoptosis: A potential mechanism of immune evasion. Nat. Med..

[23] Zhu J., Chen L., Zou L., Yang P., Wu R., Mao Y., Zhou H., Li R., Wang K., Wang W. (2014). miR-20b, -21, and -130b inhibit PTEN expression resulting in B7-H1 over-expression in advanced colorectal cancer. Hum. Immunol..

[24] Yang Y., Yang J.J., Tao H., Jin W.S. (2015). MicroRNA-21 controls hTERT via PTEN in human colorectal cancer cell proliferation. J. Physiol. Biochem..

[25] Yamamichi N., Shimomura R., Inada K., Sakurai K., Haraguchi T., Ozaki Y., Fujita S., Mizutani T., Furukawa C., Fujishiro M. (2009). Locked nucleic acid in situ hybridization analysis of miR-21 expression during colorectal cancer development. Clin. Cancer Res..

[26] Oue N., Anami K., Schetter A.J., Moehler M., Okayama H., Khan M.A., Bowman E.D., Mueller A., Schad A., Shimomura M. (2014). High miR-21 expression from FFPE tissues is associated with poor survival and response to adjuvant chemotherapy in colon cancer. Int. J. Cancer.

[27] Chen T., Yao L.Q., Shi Q., Ren Z., Ye L.C., Xu J.M., Zhou P.H., Zhong Y.S. (2014). MicroRNA-31 contributes to colorectal cancer development by targeting factor inhibiting HIF-1α (FIH-1). Cancer Biol. Ther..

[28] Xu R.S., Wu X.D., Zhang S.Q., Li C.F., Yang L., Li D.D., Zhang B.G., Zhang Y., Jin J.P., Zhang B. (2013). The tumor suppressor gene RhoBTB1 is a novel target of miR-31 in human colon cancer. Int. J. Oncol..

[29] Sun D., Yu F., Ma Y., Zhao R., Chen X., Zhu J., Zhang C.Y., Chen J., Zhang J. (2013). MicroRNA-31 activates the RAS pathway and functions as an oncogenic MicroRNA in human colorectal cancer by repressing RAS p21 GTPase activating protein 1 (RASA1). J. Biol. Chem..

[30] Zhang G., Zhou H., Xiao H., Liu Z., Tian H., Zhou T. (2014). MicroRNA-92a functions as an oncogene in colorectal cancer by targeting PTEN. Dig. Dis. Sci..

[31] Zhou T., Zhang G., Liu Z., Xia S., Tian H. (2013). Overexpression of miR-92a correlates with tumor metastasis and poor prognosis in patients with colorectal cancer. Int. J. Colorectal Dis..

[32] Tang W., Zhu Y., Gao J., Fu J., Liu C., Liu Y., Song C., Zhu S., Leng Y., Wang G. (2014). MicroRNA-29a promotes colorectal cancer metastasis by regulating matrix metalloproteinase 2 and E-cadherin via KLF4. Br. J. Cancer.

[33] Wu W., Yang J., Feng X., Wang H., Ye S., Yang P., Tan W., Wei G., Zhou Y. (2013). MicroRNA-32 (miR-32) regulates phosphatase and tensin homologue (PTEN) expression and promotes growth, migration, and invasion in colorectal carcinoma cells. Mol. Cancer.

[34] Huang Z., Huang S., Wang Q., Liang L., Ni S., Wang L., Sheng W., He X., Du X. (2011). MicroRNA-95 promotes cell proliferation and targets sorting Nexin 1 in human colorectal carcinoma. Cancer Res..

[35] Gao F., Wang W. (2015). MicroRNA-96 promotes the proliferation of colorectal cancer cells and targets tumor protein p53 inducible nuclear protein 1, forkhead box protein O1 (FOXO1) and FOXO3a. Mol. Med. Rep..

[36] Geng L., Sun B., Gao B., Wang Z., Quan C., Wei F., Fang X.D. (2014). MicroRNA-103 promotes colorectal cancer by targeting tumor suppressor DICER and PTEN. Int. J Mol. Sci..

[37] Ji D., Chen Z., Li M., Zhan T., Yao Y., Zhang Z., Xi J., Yan L., Gu J. (2014). MicroRNA-181a promotes tumor growth and liver metastasis in colorectal cancer by targeting the tumor suppressor WIF-1. Mol. Cancer.

[38] Wei Z., Cui L., Mei Z., Liu M., Zhang D. (2014). miR-181a mediates metabolic shift in colon cancer cells via the PTEN/AKT pathway. FEBS Lett..

[39] Liu H., Du L., Wen Z., Yang Y., Li J., Wang L., Zhang X., Liu Y., Dong Z., Li W. (2013). Up-regulation of miR-182 expression in colorectal cancer tissues and its prognostic value. Int. J. Colorectal Dis..

[40] Mo J.S., Alam K.J., Kang I.H., Park W.C., Seo G.S., Choi S.C., Kim H.S., Moon H.B., Yun K.J., Chae S.C. (2015). MicroRNA 196B regulates FAS-mediated apoptosis in colorectal cancer cells. Oncotarget.

[41] Zhang J., Luo X., Li H., Yue X., Deng L., Cui Y., Lu Y. (2014). MicroRNA-223 functions as an oncogene in human colorectal cancer cells. Oncol. Rep..

[42] Tsang W.P., Kwok T.T. (2009). The miR-18a* microRNA functions as a potential tumor suppressor by targeting on K-Ras. Carcinogenesis.

[43] Zheng K., Liu W., Liu Y., Jiang C., Qian Q. (2015). MicroRNA-133a suppresses colorectal cancer cell invasion by targeting Fascin1. Oncol. Lett..

[44] Wang H., An H., Wang B., Liao Q., Li W., Jin X., Cui S., Zhang Y., Ding Y., Zhao L. (2013). miR-133a represses tumour growth and metastasis in colorectal cancer by targeting LIM and SH3 protein 1 and inhibiting the MAPK pathway. Eur. J. Cancer.

[45] Xiang K.M., Li X.R. (2014). miR-133b acts as a tumor suppressor and negatively regulates TBPL1 in colorectal cancer cells. Asian Pac. J. Cancer Prev..

[46] Duan F.T., Qian F., Fang K., Lin K.Y., Wang W.T., Chen Y.Q. (2013). miR-133b, a muscle-specific microRNA, is a novel prognostic marker that participates in the progression of human colorectal cancer via regulation of CXCR4 expression. Mol. Cancer.

[47] Zhao H.J., Ren L.L., Wang Z.H., Sun T.T., Yu Y.N., Wang Y.C., Yan T.T., Zou W., He J., Zhang Y. (2014). miR-194 deregulation contributes to colorectal carcinogenesis via targeting AKT2 pathway. Theranostics.

[48] Wang B., Shen Z.L., Gao Z.D., Zhao G., Wang C.Y., Yang Y., Zhang J.Z., Yan Y.C., Shen C., Jiang K.W. (2015). miR-194, commonly repressed in colorectal cancer, suppresses tumor growth by regulating the MAP4K4/c-Jun/MDM2 signaling pathway. Cell Cycle.

[49] Fish J.E., Santoro M.M., Morton S.U., Yu S., Yeh R.F., Wythe J.D., Ivey K.N., Bruneau B.G., Stainier D.Y., Srivastava D. (2008). miR-126 regulates angiogenic signaling and vascular integrity. Dev. Cell.

[50] Li X.M., Wang A.M., Zhang J., Yi H. (2011). Down-regulation of miR-126 expression in colorectal cancer and its clinical significance. Med. Oncol..

[51] Zhou Y., Feng X., Liu Y.L., Ye S.C., Wang H., Tan W.K., Tian T., Qiu Y.M., Luo H.S. (2013). Down-regulation of miR-126 is associated with colorectal cancer cells proliferation, migration and invasion by targeting IRS-1 via the AKT and ERK1/2 signaling pathways. PLoS ONE.

[52] Zhang Y., Wang X., Xu B., Wang B., Wang Z., Liang Y., Zhou J., Hu J., Jiang B. (2013). Epigenetic silencing of miR-126 contributes to tumor invasion and angiogenesis in colorectal cancer. Oncol. Rep..

[53] Liu Y., Zhou Y., Feng X., An P., Quan X., Wang H., Ye S., Yu C., He Y., Luo H. (2014). MicroRNA-126 functions as a tumor suppressor in colorectal cancer cells by targeting CXCR4 via the AKT and ERK1/2 signaling pathways. Int. J. Oncol..

[54] Peng H., Luo J., Hao H., Hu J., Xie S.K., Ren D., Rao B. (2014). MicroRNA-100 regulates SW620 colorectal cancer cell proliferation and invasion by targeting RAP1B. Oncol. Rep..

[55] Wang M.J., Li Y., Wang R., Wang C., Yu Y.Y., Yang L., Zhang Y., Zhou B., Zhou Z.G., Sun X.F. (2013). Downregulation of microRNA-124 is an independent prognostic factor in patients with colorectal cancer. Int. J. Colorectal Dis..

[56] Zhang J., Lu Y., Yue X., Li H., Luo X., Wang Y., Wang K., Wan J. (2013). miR-124 suppresses growth of human colorectal cancer by inhibiting STAT3. PLoS ONE.

[57] Shen K., Liang Q., Xu K., Cui D., Jiang L., Yin P., Lu Y., Li Q., Liu J. (2012). miR-139 inhibits invasion and metastasis of colorectal cancer by targeting the type I insulin-like growth factor receptor. Biochem. Pharmacol..

[58] Zhang L., Dong Y., Zhu N., Tsoi H., Zhao Z., Wu C.W., Wang K., Zheng S., Ng S.S., Chan F.K. (2014). MicroRNA-139-5p exerts tumor suppressor function by targeting NOTCH1 in colorectal cancer. Mol. Cancer.

[59] Feng Y., Zhu J., Ou C., Deng Z., Chen M., Huang W., Li L. (2014). MicroRNA-145 inhibits tumour growth and metastasis in colorectal cancer by targeting fascin-1. Br. J. Cancer.

[60] Song Y., Xu Y., Wang Z., Chen Y., Yue Z., Gao P., Xing C., Xu H. (2012). MicroRNA-148b suppresses cell growth by targeting cholecystokinin-2 receptor in colorectal cancer. Int. J. Cancer.

[61] Wang X.W., Xi X.Q., Wu J., Wan Y.Y., Hui H.X., Cao X.F. (2015). MicroRNA-206 attenuates tumor proliferation and migration involving the downregulation of NOTCH3 in colorectal cancer. Oncol. Rep..

[62] Chen D.L., Wang Z.Q., Zeng Z.L., Wu W.J., Zhang D.S., Luo H.Y., Wang F., Qiu M.Z., Wang D.S., Ren C. (2014). Identification of microRNA-214 as a negative regulator of colorectal cancer liver metastasis by way of regulation of fibroblast growth factor receptor 1 expression. Hepatology.

[63] He X., Dong Y., Wu C.W., Zhao Z., Ng S.S., Chan F.K., Sung J.J., Yu J. (2012). MicroRNA-218 inhibits cell cycle progression and promotes apoptosis in colon cancer by downregulating BMI1 polycomb ring finger oncogene. Mol. Med..

[64] Ke T.W., Hsu H.L., Wu Y.H., Chen W.T., Cheng Y.W., Cheng C.W. (2014). MicroRNA-224 suppresses colorectal cancer cell migration by targeting Cdc42. Dis. Markers.

[65] Zhao H., Dong T., Zhou H., Wang L., Huang A., Feng B., Quan Y., Jin R., Zhang W., Sun J. (2014). miR-320a suppresses colorectal cancer progression by targeting Rac1. Carcinogenesis.

[66] Sun J.Y., Huang Y., Li J.P., Zhang X., Wang L., Meng Y.L., Yan B., Bian Y.Q., Zhao J., Wang W.Z. (2012). MicroRNA-320a suppresses human colon cancer cell proliferation by directly targeting β-catenin. Biochem. Biophys. Res. Commun..

[67] Wang H., Wu J., Meng X., Ying X., Zuo Y., Liu R., Pan Z., Kang T., Huang W. (2011). MicroRNA-342 inhibits colorectal cancer cell proliferation and invasion by directly targeting DNA methyltransferase 1. Carcinogenesis.

[68] Wang Y., Tang Q., Li M., Jiang S., Wang X. (2014). MicroRNA-375 inhibits colorectal cancer growth by targeting PIK3CA. Biochem. Biophys. Res. Commun..

[69] Zhang G.J., Zhou H., Xiao H.X., Li Y., Zhou T. (2014). miR-378 is an independent prognostic factor and inhibits cell growth and invasion in colorectal cancer. BMC Cancer.

[70] Sun Y., Shen S., Liu X., Tang H., Wang Z., Yu Z., Li X., Wu M. (2014). miR-429 inhibits cells growth and invasion and regulates EMT-related marker genes by targeting Onecut2 in colorectal carcinoma. Mol. Cell. Biochem..

[71] Chai J., Wang S., Han D., Dong W., Xie C., Guo H. (2015). MicroRNA-455 inhibits proliferation and invasion of colorectal cancer by targeting RAF proto-oncogene serine/threonine-protein kinase. Tumour Boil..

[72] Ma K., Pan X., Fan P., He Y., Gu J., Wang W., Zhang T., Li Z., Luo X. (2014). Loss of miR-638 *in vitro* promotes cell invasion and a mesenchymal-like transition by influencing SOX2 expression in colorectal carcinoma cells. Mol. Cancer.

[73] Xuan Y., Yang H., Zhao L., Lau W.B., Lau B., Ren N., Hu Y., Yi T., Zhao X., Zhou S. (2015). MicroRNAs in colorectal cancer: Small molecules with big functions. Cancer Lett..

[74] Di Lena M., Travaglio E., Altomare D.F. (2013). New strategies for colorectal cancer screening. World J. Gastroenterol..

[75] Ahmed F.E., Ahmed N.C., Vos P.W., Bonnerup C., Atkins J.N., Casey M., Nuovo G.J., Naziri W., Wiley J.E., Mota H. (2013). Diagnostic microRNA markers to screen for sporadic human colon cancer in stool: I. Proof of principle. Cancer Genom. Proteom..

[76] Yau T.O., Wu C.W., Dong Y., Tang C.M., Ng S.S., Chan F.K., Sung J.J., Yu J. (2014). MicroRNA-221 and microRNA-18a identification in stool as potential biomarkers for the non-invasive diagnosis of colorectal carcinoma. Br. J. Cancer.

[77] Phua L.C., Chue X.P., Koh P.K., Cheah P.Y., Chan E.C., Ho H.K. (2014). Global fecal microRNA profiling in the identification of biomarkers for colorectal cancer screening among Asians. Oncol. Rep..

[78] Wu C.W., Ng S.S., Dong Y.J., Ng S.C., Leung W.W., Lee C.W., Wong Y.N., Chan F.K., Yu J., Sung J.J. (2012). Detection of miR-92a and miR-21 in stool samples as potential screening biomarkers for colorectal cancer and polyps. Gut.

[79] Koga Y., Yamazaki N., Yamamoto Y., Yamamoto S., Saito N., Kakugawa Y., Otake Y., Matsumoto M., Matsumura Y. (2013). Fecal miR-106a is a useful marker for colorectal cancer patients with false-negative results in immunochemical fecal occult blood test. Cancer Epidemiol. Biomark. Prev..

[80] Wu C.W., Ng S.C., Dong Y., Tian L., Ng S.S., Leung W.W., Law W.T., Yau T.O., Chan F.K., Sung J.J., Yu J. (2014). Identification of microRNA-135b in stool as a potential noninvasive biomarker for colorectal cancer and adenoma. Clin. Cancer Res..

[81] Kalimutho M., del Vecchio Blanco G., di Cecilia S., Sileri P., Cretella M., Pallone F., Federici G., Bernardini S. (2011). Differential expression of miR-144* as a novel fecal-based diagnostic marker for colorectal cancer. J. Gastroenterol..

[82] Wu X.D., Song Y.C., Cao P.L., Zhang H., Guo Q., Yan R., Diao D.M., Cheng Y., Dang C.X. (2014). Detection of miR-34a and miR-34b/c in stool sample as potential screening biomarkers for noninvasive diagnosis of colorectal cancer. Med. Oncol..

[83] Li J.M., Zhao R.H., Li S.T., Xie C.X., Jiang H.H., Ding W.J., Du P., Chen W., Yang M., Cui L. (2012). Down-regulation of fecal miR-143 and miR-145 as potential markers for colorectal cancer. Saudi Med. J..

[84] Ghanbari R., Mosakhani N., Asadi J., Nouraee N., Mowla S.J., Poustchi H., Malekzadeh R., Knuutila S. (2014). Decreased expression of fecal miR-4478 and miR-1295b-3p in early-stage colorectal cancer. Cancer Biomark..

[85] Luo X., Stock C., Burwinkel B., Brenner H. (2013). Identification and evaluation of plasma microRNAs for early detection of colorectal cancer. PLoS ONE.

[86] Pu X.X., Huang G.L., Guo H.Q., Guo C.C., Li H., Ye S., Ling S., Jiang L., Tian Y., Lin T.Y. (2010). Circulating miR-221 directly amplified from plasma is a potential diagnostic and prognostic marker of colorectal cancer and is correlated with p53 expression. J. Gastroenterol. Hepatol..

[87] Toiyama Y., Takahashi M., Hur K., Nagasaka T., Tanaka K., Inoue Y., Kusunoki M., Boland C.R., Goel A. (2013). Serum miR-21 as a diagnostic and prognostic biomarker in colorectal cancer. J. Natl. Cancer Inst..

[88] Basati G., Emami Razavi A., Abdi S., Mirzaei A. (2014). Elevated level of microRNA-21 in the serum of patients with colorectal cancer. Med. Oncol..

[89] Menendez P., Padilla D., Villarejo P., Palomino T., Nieto P., Menendez J.M., Rodriguez-Montes J.A. (2013). Prognostic implications of serum microRNA-21 in colorectal cancer. J. Surg. Oncol..

[90] Yuan D., Li K., Zhu K., Yan R., Dang C. (2015). Plasma miR-183 predicts recurrence and prognosis in patients with colorectal cancer. Cancer Biol. Ther..

[91] Zhang L., Meng L., Fan Z., Liu B., Pei Y., Zhao Z. (2014). Expression of plasma miR-106a in colorectal cancer and its clinical significance. J. Southern Med. Univ..

[92] Chen Q., Xia H.W., Ge X.J., Zhang Y.C., Tang Q.L., Bi F. (2013). Serum miR-19a predicts resistance to FOLFOX chemotherapy in advanced colorectal cancer cases. Asian Pac. J. Cancer Prev..

[93] Brunet Vega A., Pericay C., Moya I., Ferrer A., Dotor E., Pisa A., Casalots A., Serra-Aracil X., Oliva J.C., Ruiz A. (2013). MicroRNA expression profile in stage III colorectal cancer: Circulating miR-18a and miR-29a as promising biomarkers. Oncol. Rep..

[94] Yang X., Zhong J., Ji Y., Li J., Jian Y., Zhang J., Yang W. (2014). The expression and clinical significance of microRNAs in colorectal cancer detecting. Tumour Biol..

[95] Liu Y., Zhou Y., Feng X., Yang P., Yang J., An P., Wang H., Ye S., Yu C., He Y. (2014). Low expression of microRNA-126 is associated with poor prognosis in colorectal cancer. Genes Chromosomes Cancer.

[96] Chu D., Zheng J., Li J., Li Y., Zhang J., Zhao Q., Wang W., Ji G. (2014). MicroRNA-630 is a prognostic marker for patients with colorectal cancer. Tumour Biol..

[97] Yang X., Zeng Z., Hou Y., Yuan T., Gao C., Jia W., Yi X., Liu M. (2014). MicroRNA-92a as a potential biomarker in diagnosis of colorectal cancer: A systematic review and meta-analysis. PLoS ONE.

[98] Fang L., Li H., Wang L., Hu J., Jin T., Wang J., Yang B.B. (2014). MicroRNA-17-5p promotes chemotherapeutic drug resistance and tumour metastasis of colorectal cancer by repressing PTEN expression. Oncotarget.

[99] Zhao Z., He J., Zhang J., Liu M., Yang S., Li N., Li X. (2014). Dysregulated miR1254 and miR579 for cardiotoxicity in patients treated with bevacizumab in colorectal cancer. Tumour Biol..

[100] Diaz T., Tejero R., Moreno I., Ferrer G., Cordeiro A., Artells R., Navarro A., Hernandez R., Tapia G., Monzo M. (2014). Role of miR-200 family members in survival of colorectal cancer patients treated with fluoropyrimidines. J. Surg. Oncol..

[101] Zhang Y., Geng L., Talmon G., Wang J. (2015). MicroRNA-520g confers drug resistance by regulating p21 expression in colorectal cancer. J. Biol. Chem..

[102] Zhang Y., Zheng L., Huang J., Gao F., Lin X., He L., Li D., Li Z., Ding Y., Chen L. (2014). miR-124 Radiosensitizes human colorectal cancer cells by targeting PRRX1. PLoS ONE.

[103] Xue Q., Sun K., Deng H.J., Lei S.T., Dong J.Q., Li G.X. (2013). Anti-miRNA-221 sensitizes human colorectal carcinoma cells to radiation by upregulating PTEN. World J. Gastroenterol..

[104] Zhang Z., Liu X., Feng B., Liu N., Wu Q., Han Y., Nie Y., Wu K., Shi Y., Fan D. (2014). STIM1, a direct target of microRNA-185, promotes tumor metastasis and is associated with poor prognosis in colorectal cancer. Oncogene.

[105] Wu L., Yu H., Zhao Y., Zhang C., Wang J., Yue X., Yang Q., Hu W. (2015). HIF-2α mediates hypoxia-induced LIF expression in human colorectal cancer cells. Oncotarget.

[106] Lu W., Fu Z., Wang H., Feng J., Wei J., Guo J. (2014). Peroxiredoxin 2 knockdown by RNA interference inhibits the growth of colorectal cancer cells by downregulating Wnt/β-catenin signaling. Cancer Lett..

[107] Levin H.L., Moran J.V. (2011). Dynamic interactions between transposable elements and their hosts. Nat. Rev. Genet..

[108] Sato K., Siomi M.C. (2013). Piwi-interacting RNAs: biological functions and biogenesis. Essays Biochem..

[109] Assumpcao C.B., Calcagno D.Q., Araujo T.M., Batista dos Santos S.E., Ribeiro dos Santos A.K., Riggins G.J., Burbano R.R., Assumpcao P.P. (2015). The role of piRNA and its potential clinical implications in cancer. Epigenomics.

[110] Moyano M., Stefani G. (2015). PiRNA involvement in genome stability and human cancer. J. Hematol. Oncol..

[111] Cheng J., Guo J.M., Xiao B.X., Miao Y., Jiang Z., Zhou H., Li Q.N. (2011). piRNA, the new non-coding RNA, is aberrantly expressed in human cancer cells. Clin. Chim. Acta.

[112] Chu H., Xia L., Qiu X., Gu D., Zhu L., Jin J., Hui G., Hua Q., Du M., Tong N. (2015). Genetic variants in noncoding PIWI-interacting RNA and colorectal cancer risk. Cancer.

[113] Xu M.D., Qi P., Du X. (2014). Long non-coding RNAs in colorectal cancer: Implications for pathogenesis and clinical application. Mod. Pathol..

[114] Ye L.C., Zhu X., Qiu J.J., Xu J., Wei Y. (2015). Involvement of long non-coding RNA in colorectal cancer: From benchtop to bedside (Review). Oncol. Lett..

[115] Li C.H., Chen Y. (2013). Targeting long non-coding RNAs in cancers: progress and prospects. Int. J. Biochem. Cell Biol..

[116] Ling H., Vincent K., Pichler M., Fodde R., Berindan-Neagoe I., Slack F.J., Calin G.A. (2015). Junk DNA and the long non-coding RNA twist in cancer genetics. Oncogene.

[117] Xue Y., Ma G., Gu D., Zhu L., Hua Q., Du M., Chu H., Tong N., Chen J., Zhang Z., Wang M. (2015). Genome-wide analysis of long noncoding RNA signature in human colorectal cancer. Gene.

[118] Han J., Rong L.F., Shi C.B., Dong X.G., Wang J., Wang B.L., Wen H., He Z.Y. (2014). Screening of lymph nodes metastasis associated lncRNAs in colorectal cancer patients. World J Gastroenterol..

[119] Dong Y., Liang G., Yuan B., Yang C., Gao R., Zhou X. (2014). MALAT1 promotes the proliferation and metastasis of osteosarcoma cells by activating the PI3K/Akt pathway. Tumour Biol..

[120] Lai M.C., Yang Z., Zhou L., Zhu Q.Q., Xie H.Y., Zhang F., Wu L.M., Chen L.M., Zheng S.S. (2012). Long non-coding RNA MALAT-1 overexpression predicts tumor recurrence of hepatocellular carcinoma after liver transplantation. Med. Oncol..

[121] Hu L., Wu Y., Tan D., Meng H., Wang K., Bai Y., Yang K. (2015). Up-regulation of long noncoding RNA MALAT1 contributes to proliferation and metastasis in esophageal squamous cell carcinoma. J. .Exp. Clin. Cancer Res..

[122] Zheng H.T., Shi D.B., Wang Y.W., Li X.X., Xu Y., Tripathi P., Gu W.L., Cai G.X., Cai S.J. (2014). High expression of lncRNA MALAT1 suggests a biomarker of poor prognosis in colorectal cancer. Int. J. Clin. Exp. Pathol..

[123] Ji Q., Zhang L., Liu X., Zhou L., Wang W., Han Z., Sui H., Tang Y., Wang Y., Liu N. (2014). Long non-coding RNA MALAT1 promotes tumour growth and metastasis in colorectal cancer through binding to SFPQ and releasing oncogene PTBP2 from SFPQ/PTBP2 complex. Br. J. Cancer.

[124] Xu C., Yang M., Tian J., Wang X., Li Z. (2011). MALAT-1: A long non-coding RNA and its important 3′ end functional motif in colorectal cancer metastasis. Int. J. Oncol..

[125] Yang M.H., Hu Z.Y., Xu C., Xie L.Y., Wang X.Y., Chen S.Y., Li Z.G. (2015). MALAT1 promotes colorectal cancer cell proliferation/migration/invasion via PRKA kinase anchor protein 9. Biochim. Biophys. Acta.

[126] Athar M., Back J.H., Tang X., Kim K.H., Kopelovich L., Bickers D.R., Kim A.L. (2007). Resveratrol: A review of preclinical studies for human cancer prevention. Toxicol. Appl. Pharmacol..

[127] Carter L.G., D’Orazio J.A., Pearson K.J. (2014). Resveratrol and cancer: Focus on *in vivo* evidence. Endocr. Relat. Cancer.

[128] Liu B., Zhou Z., Zhou W., Liu J., Zhang Q., Xia J., Liu J., Chen N., Li M., Zhu R. (2014). Resveratrol inhibits proliferation in human colorectal carcinoma cells by inducing G1/Sphase cell cycle arrest and apoptosis through caspase/cyclinCDK pathways. Mol. Med. Rep..

[129] Ji Q., Liu X., Han Z., Zhou L., Sui H., Yan L., Jiang H., Ren J., Cai J., Li Q. (2015). Resveratrol suppresses epithelial-to-mesenchymal transition in colorectal cancer through TGF-β1/Smads signaling pathway mediated Snail/E-cadherin expression. BMC Cancer.

[130] Ji Q., Liu X., Fu X., Zhang L., Sui H., Zhou L., Sun J., Cai J., Qin J., Ren J. (2013). Resveratrol inhibits invasion and metastasis of colorectal cancer cells via MALAT1 mediated Wnt/β-catenin signal pathway. PLoS ONE.

[131] Cai B., Song X.Q., Cai J.P., Zhang S. (2014). HOTAIR: A cancer-related long non-coding RNA. Neoplasma.

[132] Hajjari M., Salavaty A. (2015). HOTAIR: An oncogenic long non-coding RNA in different cancers. Cancer Biol. Med..

[133] Cai B., Wu Z., Liao K., Zhang S. (2014). Long noncoding RNA HOTAIR can serve as a common molecular marker for lymph node metastasis: A meta-analysis. Tumour Biol..

[134] Deng Q., Sun H., He B., Pan Y., Gao T., Chen J., Ying H., Liu X., Wang F., Xu Y., Wang S. (2014). Prognostic value of long non-coding RNA HOTAIR in various cancers. PLoS ONE.

[135] Yao Y., Li J., Wang L. (2014). Large intervening non-coding RNA HOTAIR is an indicator of poor prognosis and a therapeutic target in human cancers. Int. J. Mol. Sci..

[136] Wang S., Wang Z. (2015). Prognostic value of long noncoding RNA HOTAIR in digestive system malignancies. J. Gastroenterol. Hepatol..

[137] Xue Y., Gu D., Ma G., Zhu L., Hua Q., Chu H., Tong N., Chen J., Zhang Z., Wang M. (2015). Genetic variants in lncRNA HOTAIR are associated with risk of colorectal cancer. Mutagenesis.

[138] Kogo R., Shimamura T., Mimori K., Kawahara K., Imoto S., Sudo T., Tanaka F., Shibata K., Suzuki A., Komune S. (2011). Long noncoding RNA HOTAIR regulates polycomb-dependent chromatin modification and is associated with poor prognosis in colorectal cancers. Cancer Res..

[139] Wu Z.H., Wang X.L., Tang H.M., Jiang T., Chen J., Lu S., Qiu G.Q., Peng Z.H., Yan D.W. (2014). Long non-coding RNA HOTAIR is a powerful predictor of metastasis and poor prognosis and is associated with epithelial-mesenchymal transition in colon cancer. Oncol. Rep..

[140] Svoboda M., Slyskova J., Schneiderova M., Makovicky P., Bielik L., Levy M., Lipska L., Hemmelova B., Kala Z., Protivankova M. (2014). HOTAIR long non-coding RNA is a negative prognostic factor not only in primary tumors, but also in the blood of colorectal cancer patients. Carcinogenesis.

[141] Lustig O., Ariel I., Ilan J., Lev-Lehman E., De-Groot N., Hochberg A. (1994). Expression of the imprinted gene H19 in the human fetus. Mol. Reprod. Dev..

[142] Cui H., Onyango P., Brandenburg S., Wu Y., Hsieh C.L., Feinberg A.P. (2002). Loss of imprinting in colorectal cancer linked to hypomethylation of H19 and IGF2. Cancer Res..

[143] Tian F., Tang Z., Song G., Pan Y., He B., Bao Q., Wang S. (2012). Loss of imprinting of IGF2 correlates with hypomethylation of the H19 differentially methylated region in the tumor tissue of colorectal cancer patients. Mol. Med. Rep..

[144] Matouk I.J., Raveh E., Abu-lail R., Mezan S., Gilon M., Gershtain E., Birman T., Gallula J., Schneider T., Barkali M., Richler C. (2014). Oncofetal H19 RNA promotes tumor metastasis. Biochim. Biophys. Acta.

[145] Tsang W.P., Ng E.K., Ng S.S., Jin H., Yu J., Sung J.J., Kwok T.T. (2010). Oncofetal H19-derived miR-675 regulates tumor suppressor RB in human colorectal cancer. Carcinogenesis.

[146] Nissan A., Stojadinovic A., Mitrani-Rosenbaum S., Halle D., Grinbaum R., Roistacher M., Bochem A., Dayanc B.E., Ritter G., Gomceli I. (2012). Colon cancer associated transcript-1: A novel RNA expressed in malignant and pre-malignant human tissues. Int. J. Cancer..

[147] Alaiyan B., Ilyayev N., Stojadinovic A., Izadjoo M., Roistacher M., Pavlov V., Tzivin V., Halle D., Pan H., Trink B. (2013). Differential expression of colon cancer associated transcript1 (CCAT1) along the colonic adenoma-carcinoma sequence. BMC Cancer.

[148] Kam Y., Rubinstein A., Naik S., Djavsarov I., Halle D., Ariel I., Gure A.O., Stojadinovic A., Pan H., Tsivin V. (2014). Detection of a long non-coding RNA (CCAT1) in living cells and human adenocarcinoma of colon tissues using FIT-PNA molecular beacons. Cancer Lett..

[149] Xiang J.F., Yin Q.F., Chen T., Zhang Y., Zhang X.O., Wu Z., Zhang S., Wang H.B., Ge J., Lu X. (2014). Human colorectal cancer-specific CCAT1-L lncRNA regulates long-range chromatin interactions at the MYC locus. Cell Res..

[150] Ling H., Spizzo R., Atlasi Y., Nicoloso M., Shimizu M., Redis R.S., Nishida N., Gafa R., Song J., Guo Z. (2013). CCAT2, a novel noncoding RNA mapping to 8q24, underlies metastatic progression and chromosomal instability in colon cancer. Genome Res..

[151] Redis R.S., Sieuwerts A.M., Look M.P., Tudoran O., Ivan C., Spizzo R., Zhang X., de Weerd V., Shimizu M., Ling H. (2013). CCAT2, a novel long non-coding RNA in breast cancer: Expression study and clinical correlations. Oncotarget.

[152] Qiu M., Xu Y., Yang X., Wang J., Hu J., Xu L., Yin R. (2014). CCAT2 is a lung adenocarcinoma-specific long non-coding RNA and promotes invasion of non-small cell lung cancer. Tumour Biol..

[153] Qi P., Xu M.D., Ni S.J., Huang D., Wei P., Tan C., Zhou X.Y., Du X. (2013). Low expression of LOC285194 is associated with poor prognosis in colorectal cancer. J. Transl. Med..

[154] Liu Q., Huang J., Zhou N., Zhang Z., Zhang A., Lu Z., Wu F., Mo Y.Y. (2013). LncRNA loc285194 is a p53-regulated tumor suppressor. Nucleic Acids Res..

[155] Sana J., Hankeova S., Svoboda M., Kiss I., Vyzula R., Slaby O. (2012). Expression levels of transcribed ultraconserved regions uc.73 and uc.388 are altered in colorectal cancer. Oncology.

[156] Wang G., Li Z., Zhao Q., Zhu Y., Zhao C., Li X., Ma Z., Li X., Zhang Y. (2014). LincRNA-p21 enhances the sensitivity of radiotherapy for human colorectal cancer by targeting the Wnt/β-catenin signaling pathway. Oncol. Rep..

[157] Zhai H., Fesler A., Schee K., Fodstad O., Flatmark K., Ju J. (2013). Clinical significance of long intergenic noncoding RNA-p21 in colorectal cancer. Clin. Colorectal Cancer.

[158] Yin D., He X., Zhang E., Kong R., De W., Zhang Z. (2014). Long noncoding RNA GAS5 affects cell proliferation and predicts a poor prognosis in patients with colorectal cancer. Med. Oncol..

[159] Qi P., Xu M.D., Ni S.J., Shen X.H., Wei P., Huang D., Tan C., Sheng W.Q., Zhou X.Y., Du X. (2015). Down-regulation of ncRAN, a long non-coding RNA, contributes to colorectal cancer cell migration and invasion and predicts poor overall survival for colorectal cancer patients. Mol. Carcinog..

[160] Yan B., Gu W., Yang Z., Gu Z., Yue X., Gu Q., Liu L. (2014). Downregulation of a long noncoding RNA-ncRuPAR contributes to tumor inhibition in colorectal cancer. Tumour Biol..

[161] Yin D.D., Liu Z.J., Zhang E., Kong R., Zhang Z.H., Guo R.H. (2015). Decreased expression of long noncoding RNA MEG3 affects cell proliferation and predicts a poor prognosis in patients with colorectal cancer. Tumour Biol..

[162] Shi D., Zheng H., Zhuo C., Peng J., Li D., Xu Y., Li X., Cai G., Cai S. (2014). Low expression of novel lncRNA RP11-462C24.1 suggests a biomarker of poor prognosis in colorectal cancer. Med. Oncol..

[163] Li L., Sun R., Liang Y., Pan X., Li Z., Bai P., Zeng X., Zhang D., Zhang L., Gao L. (2013). Association between polymorphisms in long non-coding RNA PRNCR1 in 8q24 and risk of colorectal cancer. J. Exp. Clin. Cancer Res..

[164] Takahashi Y., Sawada G., Kurashige J., Uchi R., Matsumura T., Ueo H., Takano Y., Eguchi H., Sudo T., Sugimachi K. (2014). Amplification of PVT-1 is involved in poor prognosis via apoptosis inhibition in colorectal cancers. Br. J. Cancer.

[165] Ellis B.C., Graham L.D., Molloy P.L. (2014). CRNDE, a long non-coding RNA responsive to insulin/IGF signaling, regulates genes involved in central metabolism. Biochim. Biophys. Acta.

[166] Graham L.D., Pedersen S.K., Brown G.S., Ho T., Kassir Z., Moynihan A.T., Vizgoft E.K., Dunne R., Pimlott L., Young G.P. (2011). Colorectal neoplasia differentially expressed (CRNDE), a novel gene with elevated expression in colorectal adenomas and adenocarcinomas. Genes Cancer.

[167] Matouk I.J., Abbasi I., Hochberg A., Galun E., Dweik H., Akkawi M. (2009). Highly upregulated in liver cancer noncoding RNA is overexpressed in hepatic colorectal metastasis. Eur. J. Gastroenterol. Hepatol..

[168] Ge X., Chen Y., Liao X., Liu D., Li F., Ruan H., Jia W. (2013). Overexpression of long noncoding RNA PCAT-1 is a novel biomarker of poor prognosis in patients with colorectal cancer. Med. Oncol..

[169] Guo Q., Zhao Y., Chen J., Hu J., Wang S., Zhang D., Sun Y. (2014). BRAF-activated long non-coding RNA contributes to colorectal cancer migration by inducing epithelial-mesenchymal transition. Oncol. Lett..

[170] Shi Y., Liu Y., Wang J., Jie D., Yun T., Li W., Yan L., Wang K., Feng J. (2015). Downregulated Long noncoding RNA BANCR promotes the proliferation of colorectal cancer cells via downregualtion of p21 expression. PLoS ONE.

[171] Han Y., Yang Y.N., Yuan H.H., Zhang T.T., Sui H., Wei X.L., Liu L., Huang P., Zhang W.J., Bai Y.X. (2014). UCA1, a long non-coding RNA up-regulated in colorectal cancer influences cell proliferation, apoptosis and cell cycle distribution. Pathology.

[172] Iguchi T., Uchi R., Nambara S., Saito T., Komatsu H., Hirata H., Ueda M., Sakimura S., Takano Y., Kurashige J. (2015). A long noncoding RNA, lncRNA-ATB, is involved in the progression and prognosis of colorectal cancer. Anticancer Res..

[173] Qiu J.J., Yan J.B. (2015). Long non-coding RNA LINC01296 is a potential prognostic biomarker in patients with colorectal cancer. Tumour Biol..

[174] Ma Y., Yang Y., Wang F., Moyer M.P., Wei Q., Zhang P., Yang Z., Liu W., Zhang H., Chen N. (2015). Long non-coding RNA CCAL regulates colorectal cancer progression by activating Wnt/β-catenin signalling pathway via suppression of activator protein 2α. Gut.

[175] Kiss-Laszlo Z., Henry Y., Bachellerie J.P., Caizergues-Ferrer M., Kiss T. (1996). Site-specific ribose methylation of preribosomal RNA: A novel function for small nucleolar RNAs. Cell.

[176] Kiss-Laszlo Z., Henry Y., Kiss T. (1998). Sequence and structural elements of methylation guide snoRNAs essential for site-specific ribose methylation of pre-rRNA. EMBO J..

[177] Kiss A.M., Jady B.E., Bertrand E., Kiss T. (2004). Human box H/ACA pseudouridylation guide RNA machinery. Mol. Cell. Biol..

[178] McMahon M., Contreras A., Ruggero D. (2015). Small RNAs with big implications: New insights into H/ACA snoRNA function and their role in human disease. Wiley Interdiscip. Rev. RNA.

[179] Appaiah H.N., Goswami C.P., Mina L.A., Badve S., Sledge G.W., Liu Y., Nakshatri H. (2011). Persistent upregulation of U6:SNORD44 small RNA ratio in the serum of breast cancer patients. Breast Cancer Res..

[180] Dong X.Y., Rodriguez C., Guo P., Sun X., Talbot J.T., Zhou W., Petros J., Li Q., Vessella R.L., Kibel A.S. (2008). SnoRNA U50 is a candidate tumor-suppressor gene at 6q14.3 with a mutation associated with clinically significant prostate cancer. Hum. Mol. Genet..

[181] Gao L., Ma J., Mannoor K., Guarnera M.A., Shetty A., Zhan M., Xing L., Stass S.A., Jiang F. (2015). Genome-wide small nucleolar RNA expression analysis of lung cancer by next-generation deep sequencing. Int. J. Cancer.

[182] Xu G., Yang F., Ding C.L., Zhao L.J., Ren H., Zhao P., Wang W., Qi Z.T. (2014). Small nucleolar RNA 113-1 suppresses tumorigenesis in hepatocellular carcinoma. Mol. Cancer.

[183] Thorenoor N., Slaby O. (2015). Small nucleolar RNAs functioning and potential roles in cancer. Tumour Biol..

[184] Nallar S.C., Kalvakolanu D.V. (2013). Regulation of snoRNAs in cancer: Close encounters with interferon. J. Interferon Cytokine Res..

[185] Gee H.E., Buffa F.M., Camps C., Ramachandran A., Leek R., Taylor M., Patil M., Sheldon H., Betts G., Homer J. (2011). The small-nucleolar RNAs commonly used for microRNA normalisation correlate with tumour pathology and prognosis. Br. J. Cancer.

[186] Krell J., Frampton A.E., Mirnezami R., Harding V., De Giorgio A., Roca Alonso L., Cohen P., Ottaviani S., Colombo T., Jacob J. (2014). Growth arrest-specific transcript 5 associated snoRNA levels are related to p53 expression and DNA damage in colorectal cancer. PLoS ONE.

[187] Ferreira H.J., Heyn H., Moutinho C., Esteller M. (2012). CpG island hypermethylation-associated silencing of small nucleolar RNAs in human cancer. RNA Biol..

[188] Valadkhan S. (2005). snRNAs as the catalysts of pre-mRNA splicing. Curr. Opin. Chem. Biol..

[189] Baraniskin A., Nopel-Dunnebacke S., Ahrens M., Jensen S.G., Zollner H., Maghnouj A., Wos A., Mayerle J., Munding J., Kost D. (2013). Circulating U2 small nuclear RNA fragments as a novel diagnostic biomarker for pancreatic and colorectal adenocarcinoma. Int. J. Cancer.

[190] Baraniskin A., Nopel-Dunnebacke S., Schumacher B., Gerges C., Bracht T., Sitek B., Meyer H.E., Gerken G., Dechene A., Schlaak J.F. (2014). Analysis of U2 small nuclear RNA fragments in the bile differentiates cholangiocarcinoma from primary sclerosing cholangitis and other benign biliary disorders. Dig. Dis. Sci..

[191] Memczak S., Jens M., Elefsinioti A., Torti F., Krueger J., Rybak A., Maier L., Mackowiak S.D., Gregersen L.H., Munschauer M. (2013). Circular RNAs are a large class of animal RNAs with regulatory potency. Nature.

[192] Hansen T.B., Jensen T.I., Clausen B.H., Bramsen J.B., Finsen B., Damgaard C.K., Kjems J. (2013). Natural RNA circles function as efficient microRNA sponges. Nature.

[193] Chen L.L., Yang L. (2015). Regulation of circRNA biogenesis. RNA Biol..

[194] Zhang X.O., Wang H.B., Zhang Y., Lu X., Chen L.L., Yang L. (2014). Complementary sequence-mediated exon circularization. Cell.

[195] Li P., Chen S., Chen H., Mo X., Li T., Shao Y., Xiao B., Guo J. (2015). Using circular RNA as a novel type of biomarker in the screening of gastric cancer. Clin. Chim. Acta.

[196] Hansen T.B., Kjems J., Damgaard C.K. (2013). Circular RNA and miR-7 in cancer. Cancer Res..

[197] Li F., Zhang L., Li W., Deng J., Zheng J., An M., Lu J., Zhou Y. (2015). Circular RNA ITCH has inhibitory effect on ESCC by suppressing the Wnt/β-catenin pathway. Oncotarget.

[198] Bachmayr-Heyda A., Reiner A.T., Auer K., Sukhbaatar N., Aust S., Bachleitner-Hofmann T., Mesteri I., Grunt T.W., Zeillinger R. (2015). Correlation of circular RNA abundance with proliferation—Exemplified with colorectal and ovarian cancer, idiopathic lung fibrosis, and normal human tissues. Sci. Rep..

[199] Sharp S.J., Schaack J., Cooley L., Burke D.J., Soll D. (1985). Structure and transcription of eukaryotic tRNA genes. CRC Crit. Rev. Biochem..

[200] Thompson D.M., Parker R. (2009). Stressing out over tRNA cleavage. Cell.

[201] Goodarzi H., Liu X., Nguyen H.C., Zhang S., Fish L., Tavazoie S.F. (2015). Endogenous tRNA-derived fragments suppress breast cancer progression via YBX1 displacement. Cell.

